# Implementing Locally Sourced Food Vouchers and Unrestricted Cash Transfers for Moderate Acute Malnutrition Management in Ethiopia: Implementation Lessons from the VIMAM Trial

**DOI:** 10.3390/nu18213633

**Published:** 2026-10-31

**Authors:** Seifu Hagos Gebreyesus, Feven Hailu Tesfaye, Yakob Desalegn Nigatu, Meron Tamirat Fule, Eskeziaw Abebe Kassahun, Arsema Abebe, Genet Kiflemariam Paulos, Samson Gebremedhin, Hamid Yimam Hassen, Mufaro Chiriga, Kemeria Barsenga, Aweke Kebede, Tafara Ndumiyana, Mutinta Hambayi, Charles Uzodimma Nwokorie, Robert Ackatia-Armah

**Affiliations:** 1 Department of Nutrition and Dietetics, School of Public Health, College of Health Sciences, Addis Ababa University, Addis Ababa P.O. Box 1176, Ethiopiaeskeziaw02@gmail.com (E.A.K.);; 2 Department of Family Medicine & Population Health, Faculty of Medicine and Health Sciences, University of Antwerp, 2610 Antwerp, Belgium; 3 World Food Programme, Addis Ababa P.O. Box 25584, Ethiopia; mufchiriga@gmail.com (M.C.);

**Keywords:** MAM, moderate acute malnutrition, cash transfer, food voucher, implementation research, Ethiopia

## Abstract

**Background:** This paper documents implementation lessons from a trial evaluating locally sourced food vouchers and unrestricted cash transfers for the management of moderate acute malnutrition (MAM) among children aged 6–59 months and pregnant and breastfeeding women (PBW) in Ethiopia. **Methods:** Implementation lessons were synthesized from program documentation, routine monitoring data, supportive supervision reports, field observations, and complementary qualitative and operational studies conducted throughout the trial. Findings were examined to identify implementation bottlenecks, adaptations, and lessons across different levels. **Results:** The trial enrolled a total of 6636 participants across 102 health-post clusters, comprising 3296 children aged 6–59 months and 3340 pregnant and breastfeeding women. Voucher redemption and cash collection rates ranged from 70.2% to 88.6% across the two market-based modalities, indicating generally high transfer access, although incomplete redemption or collection remained, particularly in the cash arm. The modalities were implemented in purposively selected settings with functional markets and access to financial and digital infrastructure, supported by substantial trial-specific implementation inputs. Locally sourced food vouchers enabled households to access a defined nutritious food basket adapted to local food availability but required continuous market monitoring, retailer engagement, and adherence to basket specifications. Unrestricted cash transfers provided greater household choice and purchasing flexibility but were influenced by market conditions, price fluctuations, payment processes, and intra-household decision-making. Implementation bottlenecks included frontline health worker workload and turnover, digital system delays, shortages of cash and voucher cards, retailer non-compliance, financial service delivery challenges, and household barriers such as long travel distances, limited storage capacity, and intra-household food sharing. Adaptive management included refresher training and supportive supervision, strengthened coordination with digital and financial service providers, improved supply planning, enhanced retailer monitoring, and targeted social and behavior change communication. **Conclusions:** The implementation experience suggests that market-based modalities can be delivered through routine health-system structures when adequate market, digital, financial, and implementation capacity is available. However, these findings are based on purposively selected study settings and substantial implementation support and should not be interpreted as evidence of feasibility or sustainability at scale. Phased and context-specific scale-up is recommended, accompanied by continued assessment under routine program conditions and across settings with varying levels of market and health-system capacity.

## 1. Introduction

Acute malnutrition remains a major global public health challenge and a significant barrier to achieving the 2030 Sustainable Development Goals. Globally, an estimated 45.2 million children under five years of age were affected by wasting, with Africa accounting for more than one quarter of all affected children. Acute malnutrition contributes to nearly half of all deaths among children under five by increasing their susceptibility to infectious diseases, particularly in low- and middle-income countries [1,2]. The burden is also substantial among pregnant and breastfeeding women (PBW), especially in food-insecure and humanitarian settings. In 2022, an estimated 6.9 million pregnant and breastfeeding women and adolescent girls suffered from acute malnutrition, most of whom were affected by food and nutrition crises, placing them at increased risk of maternal morbidity, adverse birth outcomes, and poor infant health [3,4].

Sub-Saharan Africa carries a substantial burden of maternal undernutrition, with a high proportion of women entering pregnancy with inadequate nutritional status. The burden is particularly concentrated among women living in rural areas, where poverty, food insecurity, limited dietary diversity, and reduced access to health and nutrition services contribute to a higher risk of malnutrition [5]. Ethiopia continues to face a high burden of undernutrition, with 40% of children under five years of age stunted and approximately 5% wasted nationally [6]. Recurrent drought, conflict, population displacement, and economic instability continue to worsen food insecurity and disrupt access to essential health and nutrition services, further exacerbating acute malnutrition [7].

In Ethiopia, the management of moderate acute malnutrition (MAM) is primarily delivered through the Integrated Management of Acute Malnutrition (IMAM) platform. Under this framework, specialized nutritious foods (SNF) are provided through Targeted Supplementary Feeding Programs (TSFP), with support from the World Food Programme (WFP) and other partners [2,8,9]. Although this approach has demonstrated strong recovery outcomes and improvements in maternal and child nutrition outcomes [10,11], implementation challenges persist, including supply chain disruptions, high procurement and distribution costs, limited acceptability, and sharing or resale of commodities [12,13]. These challenges are further intensified by conflict, climate shocks, and access constraints, raising concerns about sustainability due to dependence on imported foods [14,15].

In response to these challenges, market-based approaches such as cash transfers and food vouchers are increasingly being explored as alternatives to commodity-based MAM management programs. Evidence from humanitarian and development settings, including Niger, Somalia, and Pakistan, suggests that these approaches can improve household food security, dietary diversity, and access to nutritious foods while supporting local markets and increasing beneficiary choice. The 2023 WHO guideline introduces a risk-stratified approach to the management of moderate wasting among children aged 6–59 months, with specially formulated foods prioritized in contexts where children face a higher risk of deterioration, including settings characterized by severe food insecurity or humanitarian crises. In lower-risk contexts, the guideline places greater emphasis on nutrition counseling and nutrient-dense home diets. This context-specific approach highlights the importance of considering market functionality and food security conditions when determining how nutritional support can be delivered. Where local markets are functional and nutritious foods are available, market-based approaches such as vouchers or cash may provide a potential mechanism for supporting access to nutrient-dense foods, whereas in high-risk contexts where markets are disrupted or food availability is inadequate, reliance on specialized nutritious foods may remain necessary [2]. However, evidence on how such context-specific approaches can be operationalized through routine health systems, particularly for both children and pregnant and breastfeeding women, remains limited.

The Voucher and Cash Intervention for the Management of Moderate Acute Malnutrition (VIMAM) trial addressed these operational challenges. Implemented by the WFP Ethiopia country office, in collaboration with the Federal Ministry of Health and Addis Ababa University, VIMAM was a cluster-randomized, three-arm study designed to evaluate market-based modalities within Ethiopia’s routine IMAM platform. The study was embedded across 13 districts with structurally high MAM caseloads and varying levels of baseline food insecurity, ranging from Stressed (IPC Phase 2) to Crisis and Emergency (IPC Phases 3 and 4). Health post clusters were randomly assigned to one of three arms: standard specialized nutritious food (SNF), locally sourced food vouchers, or unrestricted cash transfers.

The trial design enabled assessment of whether market-based transfers could support access to nutrient-dense foods through local markets while maintaining delivery through existing health-system structures. If shown to be effective and feasible in appropriate contexts, these modalities could help reduce pressure on centralized SNF supply chains and allow specialized commodities to be prioritized for areas where markets are not functional or where nutritional risk is highest.

Beyond assessing effectiveness and cost implications, the trial provided an opportunity to document the implementation requirements for introducing market-based approaches into routine nutrition services. Operational feasibility in this paper refers to the ability to deliver the assigned modality through existing health, market, digital, financial, supervision, and accountability systems while maintaining participant access, transfer completion, service continuity, and implementation fidelity. This implementation-learning study synthesizes operational challenges, adaptations, enabling factors, and lessons from the VIMAM trial to inform future programs and support cautious, context-specific scale-up of market-based nutrition interventions in Ethiopia and similar settings.

## 2. Methods

### 2.1. Study Setting and Implementation Context

The VIMAM trial was implemented in collaboration with Addis Ababa University, School of Public Health, the World Food Programme (WFP) Ethiopia Country Office, and the Federal Ministry of Health of Ethiopia (FMoH), with financial support from the Foreign, Commonwealth & Development Office (FCDO), the Government of the United States of America, and the Italian Agency for Development Cooperation (AICS). The trial was embedded within Ethiopia’s routine primary health care and Integrated Management of Acute Malnutrition (IMAM) system.

The trial was conducted across 13 districts in four regions: Oromia (Fedis, Babile, and Kurfa Chele), Sidama (Shebedino and Boricha), Central Ethiopia (Kedida Gamela, East Badawacho, Lanfuro, Mareko, and Wera), and South Ethiopia (Zala, Wonago, and Humbo). The study areas represented diverse implementation contexts, with varying levels of acute food insecurity, ranging from Stressed (IPC Phase 2) conditions in parts of Sidama and Central Ethiopia to Crisis (IPC Phase 3) and localized Emergency (IPC Phase 4) conditions in pastoral, conflict-affected, and drought-affected areas [16].

Study districts were purposively selected based on high MAM caseloads and the availability of functional local markets, participating vendors, internet connectivity, and financial service providers within approximately a 7 km radius. During the preparatory phase, market functionality and readiness were assessed to determine whether local markets could support the voucher modality, including the availability and seasonal supply of nutritious foods, retailer capacity, physical accessibility, and digital and financial infrastructure. These assessments informed site selection and implementation planning.

Primary Health Care Units (PHCUs) with at least three health posts were prioritized to allow health posts within the same PHCU to be allocated to one of the three intervention arms. The selected PHCUs were organized into 34 blocks. Within each block, three health posts were randomly assigned using a transparent lottery procedure to one of three intervention arms: unrestricted cash transfer, locally sourced food voucher, or specialized nutritious food (SNF). In total, 102 health posts were included, with 34 health posts allocated to each intervention arm.

The implementation was supported through the routine health system and involved multiple actors at community, health-post, district, regional, and national levels. Health Extension Workers (HEWs) supported screening, enrolment, counseling, follow-up, and referral, while SCOPE registrars supported digital registration and transaction tracking. WFP-contracted retailers supplied foods for voucher redemption, and financial service providers facilitated cash disbursement and related financial transactions. District and regional health authorities, WFP, Addis Ababa University, and other implementation partners provided coordination, supervision, technical assistance, and implementation support.

Table 1 summarizes the key implementation actors and their primary roles across different levels of the health system.

### 2.2. Study Design and Implementation-Research Approach

The VIMAM trial was a cluster-randomized, three-arm study evaluating alternative modalities for the management of moderate acute malnutrition (MAM) within Ethiopia’s routine IMAM platform. The three intervention arms were specialized nutritious food (SNF), locally sourced food vouchers, and unrestricted cash transfers. The unit of randomization was the health-post cluster. A total of 102 health-post clusters were allocated in a 1:1:1 ratio, with 34 clusters assigned to each of the SNF, locally sourced food voucher, and unrestricted cash transfer arms. Eligible participants received the intervention allocated to the health-post cluster through which they were enrolled. The clinical trial protocol specified the cluster allocation procedures, intervention components, eligibility criteria, follow-up schedule, clinical and nutritional outcomes, and safety monitoring procedures. Participants were followed biweekly for a maximum of 16 weeks. The present manuscript draws on implementation evidence generated alongside the trial and focuses on program delivery, operational requirements, implementation barriers, adaptations, and contextual influences rather than comparative clinical effectiveness. The trial was registered with the Pan-African Clinical Trials Registry (PACTR202409775704779).

The implementation assessment used a mixed-methods approach that integrated quantitative routine monitoring data, qualitative and observational evidence, program documentation, and complementary assessments conducted during implementation. The analysis focused on how the interventions were delivered in practice, the barriers and facilitators affecting delivery, adaptations made during implementation, and factors influencing feasibility, acceptability, reach, and continuity of service delivery.

Evidence from the trial was generated throughout the implementation period from January 2025 to December 2025. Routine monitoring and program records were used to characterize operational performance and service delivery, while supportive supervision, field observations, program review processes, and complementary qualitative assessments provided information on implementation experiences and contextual factors. Findings from these sources were subsequently compared and triangulated to identify recurring implementation patterns, contextual influences, operational bottlenecks, and adaptations that informed the lessons presented in this manuscript.

The implementation assessment focused on the delivery processes and operational experience of the intervention modalities and was not intended to estimate the clinical effectiveness or nutritional equivalence of cash and voucher modalities. Clinical effectiveness outcomes of the trial are reported separately.

### 2.3. Program Theory of Change and Implementation Framework

The implementation logic underlying the VIMAM intervention was developed as a study-specific theory of change to guide interpretation of implementation experiences. The framework linked contextual readiness and implementation inputs, including functional markets, participating retailers, digital registration systems, financial service providers, trained health workers, and implementation support, to delivery of the voucher and cash modalities within routine IMAM services. These inputs were expected to enable consistent intervention delivery, beneficiary access, appropriate use of the transfers, and functioning monitoring and feedback systems. These implementation processes were expected to contribute to improved reach, acceptability, continuity, and integration of the modalities within routine MAM services.

Contextual factors, including market conditions, food availability and prices, household access, workforce capacity, digital connectivity, and financial service performance, were considered potential modifiers of implementation. Findings from routine monitoring, supervision, qualitative assessments, and program documentation were used to examine how these pathways operated in practice and how implementation adaptations influenced delivery (Figure 1).

### 2.4. Study Population and Eligibility

The study population comprised children aged 6–59 months with moderate acute malnutrition (MAM) and pregnant and breastfeeding women (PBW) who met the eligibility criteria for community-based management of acute malnutrition. A total of 6636 participants were enrolled across 102 health-post clusters, comprising 3296 children aged 6–59 months and 3340 pregnant and breastfeeding women. Among the children, 1117 were enrolled in the SNF arm, 1128 in the unrestricted cash arm, and 1051 in the voucher arm. Among pregnant and breastfeeding women, 1133 were enrolled in the SNF arm, 1143 in the unrestricted cash arm, and 1064 in the voucher arm.

#### 2.4.1. Admission Criteria

Participants were eligible for enrollment if they met the following criteria:

• Children aged 6–59 months: mid-upper arm circumference (MUAC) ≥ 11.5 cm and < 12.5 cm, absence of bilateral pitting oedema, and clinical stability.
• Pregnant and breastfeeding women: pregnant women in any trimester and breastfeeding women up to six months postpartum with MUAC < 23.0 cm who met the national eligibility criteria for community-based management of acute malnutrition.

#### 2.4.2. Follow-Up and Exit Criteria

Participants in all three intervention arms were followed biweekly by health extension workers (HEWs) for a maximum treatment period of four months (16 weeks), corresponding to up to eight follow-up visits. Follow-up visits included assessments of nutritional recovery, treatment progress, adherence, and clinical safety.

Participants exited the program through one of three predefined pathways: (1) recovery, upon meeting the predefined nutritional recovery criteria; (2) transfer or referral, if they developed bilateral pitting oedema or another condition requiring referral to a higher level of care; or (3) default or loss to follow-up, defined as missing two consecutive scheduled follow-up visits.

### 2.5. Intervention Modalities Pathway

The three modalities were delivered at the same frequency and over the same intervention period and were accompanied by common services, including community mobilization and screening, enrolment, routine nutritional monitoring, standardized social and behavioral change communication (SBCC), and referral of participants with complications. Delivery was primarily facilitated by Health Extension Workers (HEWs), with digital registration and transaction management supported by SCOPE registrars and financial or market actors, as applicable.

#### 2.5.1. Specialized Nutritious Food (SNF) Intervention Pathway

Participants assigned to the SNF arm received the standard targeted supplementary feeding regimen. Eligible participants were enrolled at health posts by HEWs following standard MAM treatment procedures. Children aged 6–59 months received one 100 g sachet of ready-to-use supplementary food (RUSF) per day, equivalent to 15 sachets for each two-week treatment period. RUSF was provided ready to use and did not require cooking or additional preparation. Pregnant and breastfeeding women (PBW) received two packs (3 kg) of Corn-Soya Blend Plus (CSB++) per two-week treatment period, which were prepared as porridge before consumption. The prescribed SNF rations were provided biweekly at health posts by HEWs as part of routine MAM treatment.

#### 2.5.2. Locally Sourced Food Voucher Intervention Pathway

Following community-based screening and confirmation of MAM eligibility at the health post, participants assigned to the voucher arm were enrolled in the program and registered in the Supply Chain Operations Digital Platform (SCOPE). SCOPE was configured to support participant registration, biometric verification, voucher entitlement management, transaction tracking, and reconciliation of retailer payments.

Participants were followed up biweekly, and a voucher entitlement was provided during each follow-up cycle. The voucher was redeemable at designated WFP-contracted retailers for a predefined basket of locally sourced, nutrient-dense foods. The food basket was developed based on local food availability and market feasibility assessments and included specified food groups along with approved alternatives within each group.

At each biweekly follow-up cycle, participants or caregivers redeemed their voucher entitlement at participating retailers. Retailers recorded eligible voucher transactions, which were subsequently reconciled and reimbursed through the program’s financial mechanism. Voucher entitlements were provided biweekly. The monetary value of the voucher was not fixed throughout the intervention period; it varied by beneficiary group, region, and implementation period. Entitlement values were periodically reviewed and adjusted using routine market monitoring data to account for food price inflation, changes in local food availability, and other market dynamics. These adjustments were intended to maintain the purchasing power of the voucher and enable participants to obtain the predefined nutritious food basket. The region and period specific voucher values are presented in Table S1.

#### 2.5.3. Unrestricted Cash Transfer Pathway

Following HEW-led community-based screening and confirmation of MAM eligibility at the health post, participants assigned to the cash arm were enrolled and registered similarly in the SCOPE platform.

Participants were followed up biweekly, consistent with the other intervention arms. At each follow-up visit, HEWs assessed MUAC, weight, and clinical status; monitored treatment progress and adherence; provided counseling on food use and meal preparation; and delivered SBCC on infant and maternal nutrition and hygiene. Admission, follow-up, and discharge criteria were identical across the SNF, voucher, and cash arms. Household visits were conducted for participants who defaulted or showed inadequate treatment progress.

At each eligible follow-up cycle, the participant’s cash transfer was authorized and processed through designated local Financial Service Providers (FSPs). Mothers or caregivers were verified using their registered beneficiary information and SCOPE identification, after which the approved transfer was disbursed through the established payment mechanism. FSPs maintained records of completed transactions and reported disbursements and operational issues to support monitoring and accountability.

HEWs supported participants in accessing the transfers and provided guidance on using the cash to improve access to nutrient-dense foods, including cereals, legumes, vegetables, fruits, and cooking oil. The cash transfer was unrestricted; participants or caregivers were not required to purchase specific foods or redeem the transfer through designated retailers.

Cash transfers were provided biweekly. The transfer value was not fixed throughout the intervention period; it varied by beneficiary group, region, and implementation period. Transfer values were periodically reviewed and adjusted based on routine monthly monitoring of food prices, availability, accessibility, and other relevant market dynamics. The adjustments were intended to preserve the purchasing power of the transfer in the context of inflation and changing market conditions. The region and period specific cash transfer values are presented in Table S2.

#### 2.5.4. Social and Behavior Change Communication (SBCC) Package

A standardized Social and Behaviour Change Communication (SBCC) package was delivered across all three intervention arms. Delivery modalities, frequency, and responsible personnel were consistent across arms, while key messages were tailored to the specific nutrition support provided in each arm. The package comprised four complementary modalities: group education, cooking demonstrations, coffee conversations, and one-to-one counseling. HEWs delivered SBCC activities during routine follow-up visits and community sessions using standardized SBCC materials and guidance.

Group education was delivered biweekly during scheduled follow-up visits at health posts. HEWs facilitated participatory sessions covering common topics, including maternal and child nutrition, dietary diversity, infant and young child feeding, food preparation and safety, hygiene, and appropriate use of the assigned intervention. Intervention-specific messages reinforced the appropriate use of the commodities or transfers. In the SNF arm, messages emphasized correct preparation and consumption of CSB++, and prevention of sharing with other household members. In the voucher arm, messages focused on selecting and preparing foods from the approved food basket, appropriate use of the voucher entitlement, food storage and handling, and prevention of sharing of voucher-purchased foods. In the cash arm, messages emphasized dietary diversity and the selection, purchase, preparation, and consumption of nutrient-dense foods. Participants were encouraged to use the cash to purchase nutritious foods, while the transfer remained unrestricted and participants were neither required nor restricted to spend it on specific foods.

Cooking demonstrations were conducted monthly, corresponding to every other biweekly follow-up visit, by HEWs. Demonstrations provided practical instruction on preparing nutrient-dense meals using foods available through each intervention. In the SNF arm, demonstrations focused on the correct preparation and use of SNF. In the voucher arm, demonstrations used foods from the approved food basket and emphasized appropriate preparation, food hygiene, and safe storage. In the cash arm, demonstrations used commonly available nutrient-dense foods that households could purchase with the cash transfer and emphasized practical preparation of diverse and nutritious meals. Across all arms, demonstrations reinforced hand hygiene, utensil cleanliness, food safety, and appropriate preparation practices.

Coffee conversations were conducted monthly as community dialogue sessions facilitated by HEWs. Sessions engaged caregivers and, where appropriate, husbands and other influential household or community members in discussions about nutrition-related practices, household decision-making, social norms, and challenges related to use of the assigned intervention. Discussions were tailored to the intervention modality and addressed appropriate use of SNF, voucher-entitled foods, or cash transfers, as applicable, as well as household food-sharing practices and barriers to accessing or using the assigned support.

One-to-one counseling was provided by HEWs biweekly during individual follow-up visits. Counseling was tailored to the nutritional needs, circumstances, and challenges of each participant.

Across all arms, HEWs conducted biweekly follow-up visits to monitor nutritional status, assess adherence, deliver SBCC messages, and refer complicated cases. Exit criteria followed national IMAM protocols based on MUAC and clinical recovery criteria. (Figure 2).

### 2.6. Adaptation of IMAM Guidelines for Food Voucher and Cash Modalities

The implementation of cash and food voucher modalities was guided by a jointly developed guideline for cash and voucher-based management of MAM in Ethiopia, developed by WFP, Addis Ababa University, and the Federal Ministry of Health. The guideline was adapted from the 2019 national IMAM protocol to incorporate market-based modalities while maintaining core clinical management components.

Adaptations were limited to delivery mechanisms and service modalities, while screening, referral pathways, follow-up schedules, and exit criteria remained consistent with standard IMAM protocols. Modifications focused on the provision of nutritional support through cash and voucher transfers, complemented by tailored SBCC activities, including counseling and cooking demonstrations, to promote appropriate use of interventions.

### 2.7. Data Sources and Evidence Synthesis

This study synthesized implementation experiences and practical lessons from multiple complementary data sources generated during implementation of the VIMAM intervention. Data were not collected specifically for this secondary synthesis. Rather, the analysis brought together data generated through the main effectiveness trial, complementary sub-studies, routine program monitoring, supportive supervision, market assessments, and program coordination and review activities conducted during the implementation period. Each complementary activity was conducted according to its respective protocol, sampling approach, and data collection procedures and addressed a specific dimension of intervention delivery, participant experience, or implementation context. All research data contributing to this implementation analysis were collected by trained data collectors or research assistants assigned to the VIMAM trial using approved data collection tools and standardized procedures for each study component. Routine program and operational evidence, including monitoring records, SCOPE transaction data, supportive supervision reports, market monitoring records, and program review documentation, was generated by the research coordinators through established program systems and subsequently compiled and reviewed by the research team.

For this paper, the available evidence was organized and synthesized across implementation domains reflecting the major components of intervention delivery. These domains included reach and participation, intervention fidelity and adherence, voucher utilization and redemption, cash transfer delivery and use, SBCC delivery, market functionality, operational systems, adaptations and corrective actions, beneficiary and provider experiences, and costs and scale-up considerations.

The synthesis followed a structured triangulation process. Quantitative data from routine monitoring systems, SCOPE records, adherence assessments, dietary assessments, market assessments, and the effectiveness trial were examined alongside qualitative evidence from implementation assessments, supportive supervision, and program review and coordination records. Evidence from different sources was compared within each implementation domain to identify areas of convergence, complementarity, and divergence.

Convergence was considered when findings from different sources indicated a consistent implementation pattern. Complementarity was identified when one source provided additional explanation or contextual detail for findings from another source. Divergence was retained when findings differed across data sources rather than being forced into a single interpretation. Where divergence occurred, findings were examined in relation to differences in timing, population or unit of observation, implementation setting, and data collection or measurement methods. This approach allowed contextual and operational explanations for apparently inconsistent findings to be considered.

The synthesis also considered the timing of implementation events. Findings were reviewed in relation to the implementation timeline to distinguish isolated or short-term operational disruptions from recurring or systemic implementation challenges. The quality and contribution of each evidence source were considered during synthesis. The assessment considered data completeness and coverage, consistency across reporting periods and implementation settings, adherence to the relevant data collection procedures, and the potential for reporting or confirmation bias. Routine and implementer-generated documentation was interpreted cautiously because it was primarily generated for program implementation and monitoring. Greater interpretive weight was given to findings corroborated across independent or methodologically distinct sources. Evidence sources were not excluded solely because of quality limitations; instead, identified limitations informed the strength and interpretation of the resulting implementation conclusions. Operational adaptations and corrective actions documented through supervision, program monitoring, and coordination processes were considered alongside the implementation challenges they addressed.

Quantitative indicators were summarized descriptively using frequencies, proportions, and other relevant summary measures, as appropriate to each data source. Qualitative findings from the implementation assessment, supportive supervision records, and program review documents were organized using domains. Initial implementation domains were defined deductively from the intervention delivery pathway and the principal operational components of the VIMAM trial. Within these domains, recurring barriers, facilitators, adaptations, and participant or provider experiences were identified and refined through comparison across evidence sources. Findings that did not fit the predefined domains were retained and used to refine the analytical framework.

The characteristics of the contributing evidence sources, including their timing, population or unit of observation, coverage or sample, purpose, key indicators, and evidence type, are presented in Table 2.

#### Data Triangulation and Synthesis Framework

A structured triangulation framework was used to integrate findings from the different data sources across the implementation domains. The framework identified the primary and complementary evidence sources for each domain and specified the aspects of implementation that were compared and synthesized. The framework used to integrate evidence across data sources and implementation domains is presented in Table 3.

## 3. Results

### 3.1. Program Design and Set-Up

#### 3.1.1. Market and Retailer Readiness

Before trial implementation, market feasibility assessments were conducted to determine whether local markets could support delivery of a nutritious food basket for the management of MAM. The assessments followed a structured process examining key factors required for program set-up, including food availability and seasonal variation, market functionality, retailer capacity and willingness to participate, physical accessibility of markets from health facilities, availability and reliability of financial service providers (FSPs), network coverage and internet connectivity, local food consumption patterns, cultural acceptability, security considerations, and monitoring feasibility.

Standardized market feasibility, retailer selection, and contracting tools were used to assess market capacity, availability of required food commodities, retailer readiness, and the availability and capacity of FSPs. Particular attention was given to the availability of foods included in the proposed voucher basket across seasons and locations. Staple cereals and pulses were generally available throughout the year, whereas the availability of nutrient-dense perishable foods, including fruits, vegetables, and animal-source foods, varied by seasonal production patterns and geographic context.

Market functionality was assessed beyond the presence of local markets and included vendor capacity, commodity diversity, storage infrastructure, price stability, supply reliability, and physical accessibility. Retailers with adequate storage facilities, reliable supply chains, and sufficient capacity to maintain stocks of required commodities were prioritized for participation. The assessment also examined retailers’ ability to respond to increased demand and identified potential supply constraints that could affect implementation. Markets were generally able to absorb increased demand without substantial price inflation; however, differences in market capacity across locations highlighted the need for phased implementation, routine market monitoring, and adaptive management to address localized shortages and seasonal fluctuations.

Where recommended food items were not consistently available, context-appropriate alternatives were identified that maintained nutritional value and local acceptability. For example, flaxseed could serve as an alternative to groundnuts or other locally available oil-rich seeds. This process ensured that the voucher basket reflected locally available foods while maintaining its intended nutritional objectives.

Retailers were selected and contracted based on predefined criteria, including possession of a valid trade license, adequate premises and storage capacity, compliance with basic hygiene and food safety requirements, and willingness and ability to maintain stocks of the required food basket. Based on the market feasibility assessment, 668 eligible vendors were identified and contracted by WFP across the 13 study districts for participation in the food voucher program.

Overall, the assessments informed food basket design, retailer selection, identification of market and supply constraints, and the operational requirements for voucher and cash delivery.

#### 3.1.2. Digital and Financial System Readiness

Implementation readiness also required establishing the digital and financial systems necessary to support delivery of both modalities. The Supply Chain Operations Digital Platform (SCOPE), a digital platform used for beneficiary management, transfer administration, and transaction monitoring, was configured to support participant registration, biometric verification, transfer management, and payment reconciliation. Partnerships with contracted retailers and financial service providers were established before program initiation to ensure readiness for service delivery.

Digital devices and applications were tested under field conditions to assess functionality, and implementation teams received practical training on digital systems, standardized operating procedures, and beneficiary data management. Pilot testing highlighted that reliable network connectivity, timely synchronization of beneficiary records, responsive technical support, and effective coordination among health services, retailers, and financial service providers were critical for ensuring uninterrupted program delivery.

#### 3.1.3. Nutrition Basket Determination

The food basket was not developed as a simple list of commodities but through a structured, evidence-informed process that balanced nutritional requirements with local food availability, affordability, cultural acceptability, and operational feasibility. The objective was to design a locally sourced food basket that could meet the increased nutritional needs of children and PBW while remaining practical for implementation within the Ethiopian context. The process comprised five key steps:

(1) Defining the Nutritional Objectives of the Basket

The first step involved defining the nutritional requirements of target population. Reference products, including Ready-to-Use Supplementary Food (RUSF) and Super Cereal Plus (SC+), were used to establish minimum requirements for energy, protein, fat, and essential micronutrients. Particular attention was given to nutrients critical for growth, recovery, and maternal nutrition, including iron, zinc, vitamin A, B vitamins, folate, calcium, and vitamin B12.

(2) Understanding Existing Dietary Gaps

The second step focused on understanding existing dietary patterns and nutrient gaps within the implementation areas. Evidence from Ethiopia’s Fill the Nutrient Gap (FNG) analysis showed that inadequate dietary diversity, rather than insufficient energy intake alone, was a major contributor to poor nutrition. Diets were predominantly based on staple cereals and oils, with limited consumption of nutrient-rich foods such as animal-source foods, fruits, and vegetables. These findings informed the basket design by emphasizing improvements in dietary quality and diversity rather than simply increasing food quantity.

(3) Evaluate the Feasibility of Meeting Nutritional Requirements Through Locally Available Foods

Cost of Diet modeling was used to assess whether locally available foods could meet the nutritional requirements of target population under different scenarios. Foods were assessed based on nutrient density, affordability, availability, seasonality, and acceptability. Different food combinations were tested using the MAM Food Calculator to assess nutrient adequacy, portion sizes, cost, market availability, and practical feasibility.

Nutrient modeling showed that combinations of locally available foods could meet most nutritional requirements for children and PBW. However, locally available foods alone were insufficient to consistently meet some micronutrient requirements, particularly iron and B vitamins, highlighting the importance of incorporating fortified foods and monitoring basket composition over time. Different combinations of locally available foods were identified and evaluated separately for children and pregnant and breastfeeding women, with the alternative food baskets and their nutritional profiles presented in Tables S3–S6.

(4) Incorporating Operational and Household Realities

The final basket design considered practical implementation constraints, including food safety, household storage capacity, preparation requirements, and potential food losses. High-protein sources such as meat and fish were excluded due to limited cold storage capacity at both retailer and household levels, while eggs were retained as the main animal-source food.

Community consultations, household acceptability testing, and cooking demonstrations were used to assess whether the selected foods were acceptable and compatible with customary household food preparation practices. Caregivers and mothers were encouraged to use the provided foods to prepare nutrient-dense porridges.

Selected food combinations were subsequently piloted to assess cooking feasibility, taste, texture, child acceptability, and compatibility with household practices. Preparation time and portioning requirements for different age groups were also assessed. Findings from focus group discussions (FGDs) and caregiver feedback informed refinements to the basket composition and preparation guidance. Participatory piloting confirmed that the proposed food combinations could be realistically prepared, consumed, and incorporated into household diets. The food trials also identified the need for clear guidance on cooking times and preparation procedures, particularly for porridge preparation.

(5) Finalizing the Basket for Different Target Groups

Nutritional requirements were assessed for different population groups, including children aged 6–23 months, 24–36 months, 37–47 months and 48 months and above, as well as pregnant and breastfeeding women. The final basket quantity was based on the needs of a four-year-old child, providing more than 80% of nutrient requirements for this age group and exceeding 100% of requirements for younger children. For women, the basket was designed to meet breastfeeding nutritional requirements, which also provide adequate support for pregnant women.

The final food basket is structured into sub-wallets of food groups including cereals, pulses, oil-seeds, vegetables, eggs, salt and oil. Within each food category, a primary food item was specified and alternatives were permitted only when primary items were unavailable due to seasonal variation or market constraints. Cereals, pulses, and oilseeds were milled into flour, and caregivers were instructed to prepare a daily nutrient-dense supplementary porridge by combining the flour with vegetables, edible oil, and one egg. Detailed specifications and estimated nutrient composition of the 15-day food basket are presented in Table 4. The detailed nutrient profiles of the final food basket for children and pregnant and breastfeeding women are presented in Tables S7 and S8.

Overall, preparatory assessments supported the development of locally adapted food baskets that balanced nutritional requirements, cultural acceptability, affordability, market availability, and operational feasibility. However, dietary assessment conducted during trial implementation identified persistent micronutrient gaps in the diets of both children and pregnant and breastfeeding women, despite the provision of the locally sourced food basket. These findings highlight that improving access to locally available foods may not be sufficient to address all micronutrient requirements and suggest that basket refinement, including the consideration of fortified foods and/or micronutrient supplementation, may be required. Continuous assessment of dietary intake, market prices, food availability, and nutrient composition should therefore be incorporated into implementation to inform future adaptation of basket composition and voucher values.

### 3.2. Program Implementation

#### 3.2.1. Digital Beneficiary Management and Voucher Delivery Systems

The SCOPE platform was used to support beneficiary registration, enrollment, voucher account management, redemption tracking, and reconciliation of payments. During implementation of the trial, we tested two approaches for managing voucher accounts and loading entitlements: a pre-load model and a post-registration model.

The pre-load model facilitated rapid access to cash and voucher entitlements but created challenges related to unused pre-funded accounts, alignment between estimated and actual caseloads, and cash and voucher-code security. The program subsequently transitioned to a post-registration model, which strengthened alignment between confirmed beneficiary registration and voucher disbursement and reduced the risk associated with unused accounts, although it introduced potential delays before first redemption. The main differences between the two approaches are summarized in Table 5.

#### 3.2.2. Food Voucher Redemption, Cash Transfer Delivery, Follow-Up, and Monitoring

Transfer delivery and monitoring procedures were established to ensure that beneficiaries in both food voucher and unrestricted cash arms received their assigned entitlements while maintaining accountability through digital and manual tracking systems. Although both modalities used program monitoring systems to track transfer delivery and completion, the processes differed according to the type of transfer.

In the Food Voucher arm, beneficiaries received QR-coded voucher cards or digital voucher identifiers that could be used to redeem the approved food basket from contracted retailers. Retailers were trained on the approved food basket composition, food quality and hygiene standards, beneficiary verification, documentation, and record-keeping requirements. During redemption, retailers provided the agreed food basket, with limited flexibility to accommodate household preferences and seasonal availability of perishable foods. Voucher transactions were recorded through the program’s digital systems and monitored to identify incomplete, unsuccessful, or irregular transactions. Where connectivity problems prevented electronic transactions, paper-based vouchers were used as a contingency mechanism, and transactions were subsequently reconciled when the digital system was restored.

Similarly, in the Unrestricted Cash arm, beneficiaries received cash through designated financial service providers. Unlike the voucher arm, cash transfers were not restricted to a predefined food basket, and beneficiaries were free to decide how to use the transferred funds. Cash delivery, receipt, and transaction completion were monitored through the financial service provider and program digital systems. Follow-up was undertaken when transactions were unsuccessful, incomplete, or could not be confirmed, and payment records were reconciled to support accountability and ensure that the intended beneficiaries received their assigned transfers.

Across modalities, transfer delivery and transaction records were routinely reviewed to identify failed transactions, system-related interruptions, and other implementation issues requiring follow-up. These monitoring and reconciliation procedures helped maintain continuity of service and accountability throughout implementation of the trial.

#### 3.2.3. Follow-Up and Monitoring at Health Facility Level

Follow-up visits were conducted every two weeks at health facilities. During these visits, Health Extension Workers (HEWs) assessed participant’s nutritional status using MUAC, while weight measurements were collected only at designated research health post locations. In addition to anthropometric monitoring, HEWs reviewed beneficiaries’ health and nutrition status and provided counseling on appropriate use of the food basket, feeding practices, and dietary behaviors.

Monitoring data were captured through both paper-based and digital systems. HEWs used standard Integrated Management of Acute Malnutrition (IMAM) registers adapted to record information related to food voucher and cash transfer, while SCOPE registrars used tablets with the SCOPE application to support digital beneficiary tracking and transaction monitoring. A coordinated approach was used whereby designated SCOPE registrars supported multiple health posts according to scheduled MAM service days.

Implementation experience highlighted that effective voucher and cash delivery required strong coordination among health workers, retailers, and digital system teams. Reliable communication, clear roles and responsibilities, functional backup mechanisms during network disruptions, and routine monitoring were essential to ensure continuity of service and maintain data quality.

#### 3.2.4. Monitoring, Supply Chain, and Accountability Systems

A comprehensive monitoring and accountability system was established to support implementation quality, safeguard program resources, and identify areas requiring corrective action. Monitoring combined routine process monitoring at health facilities and voucher redemption points with market monitoring. Monitoring was conducted with key indicators covering adherence to eligibility criteria, completeness and quality of services, beneficiary experience and waiting times, compliance with standard operating procedures (SOPs) by staff and retailers, and food quality and availability in markets.

Monitoring staff and HEWs received training on monitoring tools and protocols, supported by regular supervision visits and feedback mechanisms. These processes helped identify implementation challenges early and supported continuous program improvement.

Market monitoring focused on the availability and prices of voucher basket commodities, retailer compliance with contractual requirements, beneficiary satisfaction, and potential cases of malpractice, including short-changing, substitution with lower-quality commodities, or other forms of non-compliance.

Retailers were supervised through joint market support teams involving district authorities, relevant government offices, and technical teams, including Supply Chain, Cash-Based Transfer, Nutrition, Gender, Protection, and Monitoring and Evaluation staff. Retailers found to be engaging in fraudulent practices or repeatedly failing to comply with program requirements were subject to corrective actions, including verbal and written warnings, temporary suspension of services or enrolment, and contract termination in severe or repeated cases.

The Complaint and Feedback Mechanism (CFM) was an important component of program accountability and adaptive management. The food voucher and unrestricted cash interventions used WFP’s existing CFM system, including toll-free hotlines (6063 and 6065), to enable beneficiaries, district focal persons, and other stakeholders to report concerns, seek clarification, and provide feedback.

Monthly CFM logs documented approximately 50–80 calls from district focal persons, retailers, and beneficiaries, indicating regular use of the feedback mechanism during implementation. Complaints were resolved within an average of three days, with resolution times generally ranging from 2 to 4 days. Unresolved or high-priority cases were escalated for further action through the established CFM procedures. CFM staff were oriented on the food voucher and unrestricted cash modalities to enable them to address routine beneficiary concerns directly and refer more technical, sensitive, or fraud-related cases to the appropriate technical teams. CFM records identified recurring implementation issues, including long distances to markets, cases in which beneficiaries reported that their entitlements had already been redeemed, voucher balances not appearing at redemption points, retailer-related financial service provider transaction problems, payment delays, and fraud-related disputes. Where follow-up with the complainant was required, callers were contacted to obtain additional information or communicate the status of the reported issue. These records provided documented evidence of the identification, tracking, follow-up, escalation, and resolution of implementation-related concerns.

### 3.3. Evidence of Operational Feasibility

The clinical outcomes of the VIMAM trial and related sub-studies have been submitted for publication separately and are not presented in detail in this manuscript. This paper focuses on the implementation experience of delivering locally sourced food vouchers and unrestricted cash transfers for the management of MAM within routine program systems.

Implementation experience from the trial indicated that locally sourced food vouchers or cash transfer could be operationalized within existing health, market, and beneficiary support systems in settings where local markets were functional and a range of nutritious foods was available. The voucher modality enabled beneficiaries to access locally available foods through contracted retailers while leveraging existing systems for beneficiary registration, voucher redemption, transfer monitoring, complaint and feedback management, and transaction reconciliation. Similarly, the unrestricted cash modality was delivered through established financial service provider systems, with digital monitoring and follow-up mechanisms used to track transfer delivery and address incomplete or unsuccessful transactions.

The intervention showed potential to expand access to diverse, locally available foods while leveraging existing market systems and community structures. The findings from the trial, together with the implementation lessons presented in this paper, provide important insights into the conditions required for successful delivery of market-based approaches for MAM management.

### 3.4. Lessons and Key Challenges

This section discusses the key implementation challenges identified throughout the delivery of the VIMAM trial. It highlights the factors that influenced program implementation, describes adaptations made during implementation, and outlines the operational considerations that should be taken into account when applying similar approaches in other contexts. Table 6 summarizes the main implementation bottlenecks encountered, the adaptive management actions taken, and the lessons learned throughout implementation of the VIMAM trial in Ethiopia.

#### 3.4.1. Health Facility Readiness and Service Delivery

Health facilities served as the primary platform for participant enrolment, clinical assessment, maternal and child health services, follow-up, nutrition counseling, and delivery of program interventions. Before implementation, facility readiness assessments were conducted to evaluate infrastructure, staffing capacity, equipment availability, and essential supplies required to support trial delivery. Although most facilities met the minimum requirements for implementation, variation in operational readiness across study districts influenced early implementation. Key challenges included shortages of anthropometric equipment, registration cards and intermittent internet connectivity, particularly in remote areas, which affected participant flow, delayed registration, and constrained use of the SCOPE platform. In addition, HEWs faced significant workload pressures as they were responsible for delivering the full continuum of MAM services while managing routine health and nutrition responsibilities. Staff turnover, competing program priorities, and limited time availability affected consistency of service delivery and increased the need for additional technical support.

To address these challenges, implementation teams used adaptive management approaches, including supportive supervision, refresher training, regular mentoring, redistribution of essential equipment and supplies, and adjustment of facility workflows to improve participant flow and integrate intervention activities into routine services. Close coordination between health facility staff, research teams, and implementation partners enabled timely identification and resolution of operational bottlenecks. Additional supervisory visits were conducted in facilities requiring further support to strengthen adherence to study procedures and maintain consistency across implementation sites.

The implementation experience demonstrated that successful delivery of market-based nutrition interventions depends not only on the availability of intervention commodities and financial mechanisms but also on the readiness and capacity of health facilities to absorb additional program activities. Early assessment of facility capacity, continued investment in frontline health workers, supportive supervision, and flexible problem-solving mechanisms were critical for maintaining implementation quality across diverse settings.

#### 3.4.2. Workforce Dynamics and Protocol Fidelity: The Role of HEW

Health Extension Workers (HEWs) played a central role in implementing the VIMAM trial by linking communities with health facilities and supporting delivery of intervention activities throughout the implementation period. Implementation highlighted several workforce-related challenges that affected protocol fidelity. Initial gaps in protocol understanding, particularly related to food basket composition, pricing, digital voucher redemption procedures, and documentation requirements, were observed in settings where standardized participant reference materials were not consistently available. These knowledge gaps contributed to inconsistent caregiver counseling, incomplete reporting of retailer-related issues, and variation in implementation practices across sites. In addition, technical challenges related to anthropometric measurements and eligibility assessment, including inaccurate age determination and inconsistencies in MUAC measurement, resulted in some cases of misclassification and prolonged program participation. HEWs also faced social pressure from communities advocating for broader enrolment, creating challenges in maintaining adherence to eligibility criteria while managing community expectations.

To address these challenges, implementation teams strengthened supportive supervision, provided refresher training on intervention procedures, reinforced standardized guidance materials, and increased engagement between HEWs, research teams, and program managers. Additional efforts focused on improving measurement quality, reinforcing admission and discharge criteria, and providing regular feedback to health workers. These approaches improved consistency in implementation and supported HEWs in balancing technical requirements with community expectations.

The implementation experience demonstrated that effective implementation depended not only on initial training but also on continuous capacity building, supportive supervision, manageable workloads, and strong integration with existing health systems. Strengthening frontline health worker capacity, providing practical decision support tools, and ensuring ongoing mentorship should therefore be considered essential components of future program scale-up.

#### 3.4.3. Digital Infrastructure and SCOPE Platform

The WFP Supply Chain Operations Digital Platform (SCOPE) served as the primary digital system for beneficiary registration, verification, transfer management, and program monitoring. The platform supported biometric registration, participant tracking, voucher and cash account management, and reconciliation of cash and food voucher transfers, providing a centralized mechanism for managing beneficiaries across study sites. Implementation demonstrated several benefits of digital systems, including reduced duplication through biometric verification, improved accuracy of beneficiary records, and enhanced accountability through integration of registration and transfer management processes.

These benefits were also reflected in qualitative findings. SCOPE coordinators viewed digital registration and photo verification as important safeguards against duplicate registration and misuse. One male SCOPE coordinator explained, “The photo scan system within the SCOPE Registrar’s process effectively prevents double entry and fraud. Participants now understand they cannot send another person to collect their items.”

However, implementation also highlighted several operational challenges. In remote and resource-constrained settings, unreliable connectivity, poor lighting conditions, and limited technical infrastructure affected real-time data synchronization, resulting in participant waiting times and delays in voucher processing. Initial shortages of SCOPE registrars required rapid recruitment, and differences in staff experience and training affected consistency in system use and understanding of nutrition-related procedures. Additional challenges included PIN expiry issues and limited system functionality for identifying missed redemptions or supporting automated monitoring of program exit criteria.

Qualitative findings reinforced the importance of these technical constraints. One male nutrition focal person described how PIN expiry affected beneficiaries who attended on market days rather than on their scheduled appointment days: “Mothers and caregivers prefer to come on Thursday because it is a market day and transport is more available. Some attend on Thursday even when they were appointed on other days; as a result, their PIN expires within 24 h. When the PIN expires, another PIN has to be generated, which was challenging.” Poor connectivity was also reported as a barrier to completing registration, with one SCOPE coordinator noting, “Sometimes we need networks and are obliged to go to town to complete registrations.”

To address these challenges, implementation teams strengthened SCOPE registrar training, clarified roles and responsibilities between registrars and HEWs, increased technical support, and introduced practical solutions such as decentralized troubleshooting and mobile-based PIN extension support during market-day activities. Regular coordination between technical teams, health facilities, and service providers facilitated timely identification and resolution of operational issues and helped maintain continuity of service delivery.

The experience also highlighted the importance of adapting voucher management approaches to balance rapid access, accountability, and financial control. The initial pre-load model enabled immediate voucher redemption following enrolment but created risks related to unused pre-funded accounts, inclusion errors, and potential misuse due to reliance on estimated caseloads. Transitioning to a post-registration model strengthened program integrity by linking voucher loading to verified beneficiary registration, improving alignment between actual caseloads and financial disbursement. However, this approach introduced a short delay before first redemption, emphasizing the need for efficient registration, approval, and payment processes.

Overall, the implementation experience demonstrated that successful digital delivery requires more than functional technology. It depends on appropriate workforce planning, clear role definitions, reliable technical support, effective data management systems, and strong coordination among health services, financial service providers, and program teams. These elements are essential for maintaining both accountability and timely access to nutrition support when scaling market-based interventions.

#### 3.4.4. Retailer Engagement, Market Functionality, and Accountability

Implementation showed that successful voucher-based nutrition programs require continuous retailer engagement beyond initial training. Although retailers received pre-implementation training on voucher procedures, food basket specifications, record keeping, and reporting requirements, early implementation revealed gaps in understanding program standards. Market monitoring data showed that price fluctuations were the most frequently reported reason for retailer non-compliance (53.4%), followed by gaps in understanding recommended quantities and food basket specifications. These findings highlighted the need for ongoing refresher training, supportive supervision, and clear communication of retailer responsibilities throughout implementation.

The intervention also demonstrated the importance of functional and adaptable local markets for maintaining the nutritional quality of voucher-supported food baskets. Seasonal availability, inflation, and food price fluctuations affected retailers’ ability to consistently provide the recommended basket. Price inflation was the leading reported cause of stock-outs (51.9%), followed by supplier delivery delays and seasonal supply gaps. In some locations, rising prices were associated with retailers providing reduced quantities or substituting food items without authorization, resulting in deviations from the approved food basket. These experiences highlighted the importance of continuous market monitoring, regular price reviews, and adaptive program management to protect the nutritional objectives of the food basket while responding to changing market conditions.

Strong market oversight was also critical to implementation quality. Areas with active Market Support Groups (MSGs) and routine joint market monitoring were better able to identify retailer non-compliance and implement timely corrective actions. Market monitoring records identified prices above agreed or prevailing market levels (34.8%) and the provision of non-approved food items (30.4%) among the most frequently reported retailer malpractices. Qualitative findings provided further insight into the nature of these practices. A regional nutrition coordinator described reports of retailers retaining funds from beneficiary voucher cards, claiming that funds were unavailable, providing only part of the approved food basket, and failing to display the approved basket at the point of sale. In response, MSGs issued warnings, referred cases to relevant authorities, and conducted community awareness activities. These findings underscore the importance of active local oversight structures for identifying and responding to retailer-level deviations.

Beneficiary feedback also showed that households sometimes negotiated with retailers to obtain preferred food alternatives, which could result in deviations from the approved food basket. This highlighted the need to consider both market constraints and household preferences when designing and monitoring voucher-based interventions.

Accountability mechanisms were essential for protecting beneficiary entitlements and maintaining program integrity. Reports of transactions processed without beneficiary presence, incomplete basket delivery, and discrepancies in voucher balances demonstrated the importance of transparent redemption procedures, retailer monitoring, and effective complaint and feedback mechanisms. Routine supervision, beneficiary feedback, and coordination among program teams enabled timely identification and resolution of implementation issues, while recurring complaints informed improvements to voucher management processes.

Overall, the experience demonstrated that voucher-based nutrition programs depend on more than market availability. They require capable retailers, effective market governance, responsive monitoring systems, and strong accountability mechanisms. Continuous retailer engagement, routine market assessment, active oversight structures, and functioning complaint mechanisms were critical to maintaining program fidelity and ensuring that voucher assistance translated into improved access to nutritious foods.

#### 3.4.5. Financial Service Providers in Cash-Based Transfers

The cash transfer modality was implemented in partnership with a Financial Service Provider (FSP) responsible for processing beneficiary payments. Following verification through the SCOPE platform, eligible participants received cash transfers according to the planned payment schedule. The cash modality provided caregivers and mothers with greater flexibility to purchase nutritious foods from available local markets.

Qualitative findings reinforced the importance of accurate payroll management and data synchronization. One Health Extension Worker reported that, although payments were initially completed without major problems, payroll errors emerged after the first three months, including cases in which “names [were] missing from the payroll” despite participants having been correctly registered and submitted through SCOPE. Such discrepancies resulted in participants arriving at payment points without their names appearing on the payroll, causing frustration and requiring additional follow-up.

In addition, some financial service providers were reluctant to travel to remote health posts because of the absence of clear logistical arrangements and incentives for outreach services. This resulted in delayed arrivals at some sites, extended waiting times for caregivers and pregnant and breastfeeding women, and occasional disruption of scheduled follow-up visits. These logistical constraints were also described by an FSP branch manager, who noted that reaching some health posts was difficult because “there is no transportation facilitated to access the health posts,” requiring payment staff to travel by motorcycle to health posts located approximately 30 km from the bank. These experiences highlighted the need to plan transport and outreach arrangements as an integral part of cash transfer delivery.

Further operational challenges arose from differences between standard banking procedures and the requirements of a time-sensitive nutrition program. Occasional discrepancies between digital payment records and physical verification documents required reconciliation before disbursement, resulting in additional delays. Variation in implementation practices across sites highlighted the importance of ensuring that financial service provider staff fully understands program procedures and beneficiary service expectations.

To address these challenges, regular coordination mechanisms were established among AAU, WFP, district health offices, and financial service providers. Weekly reconciliation of beneficiary records, clarification of roles and responsibilities, dedicated communication channels, and ongoing technical support improved coordination and reduced payment delays over time. Engagement with bank managers also strengthened institutional ownership and enabled faster resolution of operational issues.

The implementation experience from the trial demonstrated that financial service providers should be considered strategic program partners rather than solely payment agents. Successful cash-based nutrition interventions require early engagement with financial institutions, clearly defined operational roles, dedicated focal points, appropriate arrangements for outreach services, and routine coordination mechanisms to ensure timely transfers and uninterrupted access to nutrition support.

#### 3.4.6. Caregivers’ Acceptability, Household Practices, and Utilization Challenges

Qualitative findings indicated that some caregivers and pregnant and breastfeeding women viewed the intervention positively and perceived improvements in their knowledge and confidence in preparing nutritious meals using locally available foods. A caregiver explained, “Earlier, we prepared by common practice and were not doing food preparation based on appropriate procedures before the demonstration. After advice and demonstration, there is a great change.” Another caregiver described sharing the knowledge gained through the intervention with others in her community: “I even share this knowledge with my neighbors, teaching them how the voucher program helps. Some neighbors now prepare food the way I learned. We have learned a lot and changed our ways.” These accounts illustrate how the intervention was perceived not only as a source of food assistance but also as an opportunity to strengthen household nutrition knowledge and feeding practices.

Despite positive perceptions, several implementation challenges affected optimal utilization of the intervention. The adherence study found that, among participants enrolled in the program, transfer access was generally high, with redemption and cash collection rates ranging from 70.2% to 88.6% across the voucher and cash modalities. Although most enrolled participants accessed their assigned transfers, incomplete redemption or collection remained, particularly for cash transfers. These findings indicate that, despite generally high utilization, operational and access-related barriers affected timely receipt of entitlements.

Physical access to markets was one such barrier. In some implementation areas, participating retailers were located beyond the intended service radius, requiring caregivers to travel long distances, in some cases more than 7 km, to redeem vouchers. Transportation costs placed an additional burden on vulnerable households, while occasional payment delays and digital system challenges resulted in repeated visits and contributed to missed appointments or interruptions in program participation.

The design of the voucher redemption cycle also created challenges for some households. The inclusion of perishable foods, particularly fruits and vegetables, within a two-week redemption cycle created difficulties for households with limited storage capacity. To reduce transport costs, many households redeemed their full allocation in a single visit, increasing the risk of early consumption or spoilage of perishable commodities. This experience highlighted the importance of aligning food basket composition, redemption schedules, and market arrangements with household storage capacity and local market conditions.

Household practices and socio-cultural factors also influenced the extent to which beneficiaries received the intended nutritional benefits. Although the food basket design accounted for expected intra-household sharing and nutrient losses during preparation, sharing of allocated foods remained common due to cultural norms around communal eating and widespread household food insecurity. As a result, foods intended for children or PBW were sometimes shared with other household members, reducing the quantity available to the target beneficiary.

In cash transfer areas, competing household priorities and limited decision-making power among some women also influenced how transfers were used. Qualitative findings illustrated how household gender dynamics could affect women’s control over cash transfers. A regional nutrition coordinator reported that, in some households, husbands took control of the cash after it was collected from the financial service provider and used it for other household or community priorities, including contributions to community gatherings. The coordinator explained: “Husbands wait for the day when the money is given, take the money from the mother as soon as she leaves the bank and spend it on their own affairs, for example, some of them drink alcohol by the money, pay for other community gatherings.” These findings illustrate how unrestricted cash provided flexibility but could also be influenced by intra-household decision-making and competing household or community demands. The implementation experience demonstrated that market-based nutrition interventions require complementary social and behavior change communication, household engagement, gender-responsive approaches, and program designs that consider real household constraints, including transport costs, food storage capacity, and intra-household decision-making dynamics.

#### 3.4.7. Social and Behavior Change Communication (SBCC)

Social and Behavioral Change Communication (SBCC) was an integral component of the VIMAM trial, supporting appropriate use of cash and voucher transfers and promoting appropriate maternal and child feeding practices. The SBCC package included group education, individual counseling, cooking demonstrations, and community coffee conversations, delivered primarily by HEWs. Integrating SBCC with market-based nutrition interventions strengthened caregiver engagement and reinforced key nutrition messages throughout implementation. Qualitative findings illustrated the practical role of these activities. One Health Extension Worker described cooking demonstrations as step-by-step sessions in which caregivers were shown how to prepare nutritious foods using locally available ingredients, including porridge, kale, oil, egg and other locally available foods.

Delivery was affected by operational challenges, including delays in the availability of SBCC materials, limited resources for cooking demonstrations, and competing HEW responsibilities, which reduced the frequency of some activities in certain districts. Participants also identified limitations in relying on verbal communication alone. A Nutrition Focal Person explained, “Providing information orally to all participants about the food voucher is difficult. Since this program is new, SBCC is important for its promotion.” Participants recommended complementing interpersonal counselling with culturally appropriate SBCC materials, including posters, banners, and radio programs in local languages, as well as engaging community and religious leaders to reinforce program messages and improve community awareness. In response to these challenges, implementation teams adapted delivery approaches through flexible scheduling, use of locally available ingredients for demonstrations, and continuous mentoring and supervision of HEWs.

The implementation experience demonstrated that SBCC is important for translating access to nutritious foods into appropriate feeding practices and supporting sustained behavior change. Future scale-up in similar setting should ensure the timely availability of standardized materials, dedicated resources for community activities, and continued support for frontline workers to deliver effective and context-appropriate SBCC. The full detailed implementation bottleneck, lesson learned and adaptive management is presented in Table S9.

## 4. Discussion

This trial contributes to the growing evidence on implementation of market-based approaches for the management of moderate acute malnutrition (MAM) by providing practical insights into delivery within routine health system settings. Evidence remains limited on how different delivery strategies interact with implementation context, health-system capacity, market functionality, and household circumstances to influence the quality and continuity of program delivery [17]. Our findings demonstrate that successful implementation depended not on the transfer modality alone, but on the readiness and coordination of the systems required to deliver it. Workforce capacity, retailer readiness, digital connectivity, financial service delivery, market conditions, and household access jointly influenced implementation. These findings reinforce the importance of systematic documentation of implementation experience during both pilot and scale-up phases, particularly where new delivery modalities are introduced through existing systems.

MAM management has traditionally relied on specialized nutritious foods (SNF), including fortified blended foods and ready-to-use supplementary foods, delivered through health system and community-based platforms. These approaches provide standardized nutrient delivery but require established systems for procurement, transportation, storage, and distribution [18]. Recent evidence suggests that locally sourced food approaches may also have potential, although their nutritional performance varies according to food type and formulation. A recent systematic review and non-inferiority meta-analysis found that locally processed foods were non-inferior to SNFs for recovery, whereas locally available foods prepared from household or community ingredients showed lower recovery rates than SNFs, highlighting the importance of food formulation and implementation context [19]. Cash and food vouchers represent alternative mechanisms for enabling households to access locally available nutritious foods and may offer greater flexibility and choice, particularly where markets are functional [20]. However, cash and vouchers should not be interpreted as nutritionally equivalent to SNF, because their nutritional contribution depends on what foods are available, purchased, and consumed by households. The present study focused on the implementation and operational delivery of these modalities rather than their nutritional equivalence or clinical effectiveness relative to SNF.

Evidence from cash-based programs indicates that implementation is influenced by program design, technical and organizational capacity, available resources, beneficiary targeting, technology, and contextual conditions [21]. Our findings extend this evidence by illustrating how these factors interacted across the delivery system during implementation of cash and voucher modalities. Staffing constraints, payment delays, digital system challenges, retailer limitations, and physical access barriers were interconnected rather than isolated implementation problems. For example, weaknesses in payroll reconciliation could delay payments, while inadequate retailer coverage increased transport burdens for households. These interdependencies suggest that scale-up readiness should be assessed across the full delivery pathway, rather than by considering individual components of a modality in isolation.

The implications for scale-up are particularly important because the trial was implemented under conditions that included purposively selected sites with relevant market, vendor, connectivity, and financial service characteristics. Delivery was further supported by research assistants, trained SCOPE registrars, contracted retailers, financial service providers, joint supportive supervision, and WFP technical assistance. These inputs helped establish and maintain the delivery systems required by the intervention. However, they also represent conditions that may not be consistently available in less-selected or lower-capacity settings. Some functions, such as SCOPE registration, retailer engagement, routine supervision, data reconciliation, and inter-partner coordination, could potentially be integrated into existing systems. Other inputs, including additional staffing, intensive technical support, and transport arrangements for outreach payments, and sustained market monitoring, may require additional resources and explicit planning. Scale-up should therefore distinguish between trial-supported functions that can be absorbed into routine systems and those that require additional financing, staffing, infrastructure, or institutional arrangements.

Community-based delivery provides an important platform for this integration. Community-based mechanisms can improve access to MAM services and provide additional opportunities for infant and young child feeding (IYCF) counseling and support. However, integration can also increase workload for frontline workers and contribute to variability in service quality [1]. Evidence from community-based acute malnutrition programs similarly emphasizes the importance of training, supervision, and implementation support, while highlighting competing demands on community health workers involved in multiple services [22]. Moreover recent evidence from Ethiopia has also demonstrated variation in MAM implementation fidelity, including gaps in follow-up and documentation when additional burden is incurred in the system [23]. In our study, the introduction of new delivery modalities similarly increased demands on Health Extension Workers (HEWs) and required refresher training, mentoring, supportive supervision, and additional implementation support. This reinforces the need to assess workforce capacity and workload before introducing additional responsibilities into routine health services.

Comparisons of program performance across settings remain challenging due to differences in indicators, reporting practices, and contextual variability. Consistent with global experiences, there is no universally agreed definition of program success in MAM management, and thresholds for coverage and adherence vary across studies. This lack of standardization limits comparability and highlights the need for harmonized indicators that are linked to meaningful nutritional outcomes [24].

Evidence showed that defining voucher food baskets, particularly the inclusion of animal-source foods, remains challenging due to storage constraints [25,26]. In our study, baskets included eggs, fruits, and vegetables, while meat or fish were not feasible because of limited storage and safety concerns at both vendor and household levels. The two-week voucher period also posed challenges, as most households redeemed the full allocation at once to reduce transport costs, leading to early consumption or spoilage of perishable foods. These findings highlight the need to balance nutritional goals with practical constraints by considering shelf-stable options, improving storage capacity, or adjusting redemption schedules.

Food voucher-based nutrition programs across settings show that success depends on strong design and implementation. In northern Cameroon, combining standardized food baskets with nutrition education and follow-up achieved high redemption and recovery rates meeting Sphere standards [27]. Similarly, programs in Haiti, Kenya, Pakistan, and the occupied Palestinian Territories improved dietary diversity through well-designed baskets, market assessments, and ongoing monitoring [28]. Our findings are consistent, showing that locally adapted baskets, structured redemption, vendor engagement, and SBCC improved access to nutritious foods. Key lessons include aligning baskets with local diets, ensuring vendor capacity, integrating with health systems, and maintaining market monitoring to preserve purchasing power. Although our study included initial market assessments and vendor agreements, inflation and vendor limitations occasionally constrained food access. Overall, context responsive design and continuous monitoring are essential for effective voucher interventions.

Global practice recommends that dietary management of MAM among children aged 6–59 months be complemented with comprehensive health assessment and appropriate management of underlying clinical conditions to enhance recovery and reduce the risk of relapse [2]. Implementation of the present study through the health system ensures that participants receive clinical assessment, treatment for identified health conditions and routine preventive services including vaccinations, antenatal and postnatal care where appropriate. Behavioral factors play a central role in determining program effectiveness across all MAM interventions. Similarly to findings from other settings, challenges related to appropriate use and sustained adherence are commonly reported. Programs that include strong SBCC components tend to demonstrate better outcomes [29]. Behavioral factors play an important role in the utilization of nutrition interventions. Similarly to findings from other settings, challenges related to appropriate use and sustained adherence were observed across the intervention modalities. Evidence from nutrition interventions indicates that adherence to the recommended basket can vary substantially across settings and is influenced by caregiver knowledge, perceptions, and other contextual factors [30]. These observations highlight the importance of integrating SBCC strategies as a core component of program design rather than as a supplementary activity.

Across different settings, evidence suggests that the choice of MAM management modality needs to be guided by local context rather than a universal delivery model. Consistent with global program experiences, our findings demonstrate that implementation requirements differ across modalities and are influenced by factors such as health system capacity, market functionality, operational infrastructure, and household circumstances. Rather than applying a single approach, programs may require adaptable delivery strategies that align modality selection and implementation design with local conditions and available systems [17].

Overall, several key lessons emerge from the comparison of our findings with other MAM programs globally. First, there is no one-size-fits-all approach to MAM management, and program effectiveness depends heavily on contextual adaptation. Second, integration with existing systems can enhance access, process adherence and sustainability but requires adequate system capacity. Third, community-based approaches are essential for improving reach but must be supported by sufficient resources and workforce capacity. Finally, sustained SBCC efforts and continuous capacity building are critical for achieving and maintaining program effectiveness.

An important implication is that implementation feasibility should not be conflated with effectiveness. The present study demonstrates the operational requirements and challenges of delivering cash and voucher modalities within a health-system platform, but it does not establish nutritional equivalence, clinical superiority, or cost-effectiveness relative to SNF. Effectiveness and cost-effectiveness findings are addressed separately in the companion analyses. Future research should examine how implementation characteristics, including market functionality, redemption, adherence, SBCC exposure, and delivery fidelity, relate to clinical and nutritional outcomes. Such evidence would help determine not only whether a modality can be delivered, but also under what implementation conditions it can achieve its intended nutritional outcomes.

Continued implementation research and systematic documentation of program experiences are needed to strengthen the evidence base for MAM management. Future research should prioritize standardized implementation indicators, determinants of program adherence and utilization, and strategies for sustaining program quality during scale-up. Strengthening knowledge sharing across programs will further inform policy and implementation.

### Strengths and Limitations

This study has several strengths. It was embedded within routine health systems and examined multiple delivery modalities, including unrestricted cash and locally sourced food vouchers, while considering the interconnected health, market, digital, and financial systems required for delivery. The use of routine monitoring data complemented by qualitative findings provided both operational and contextual perspectives on implementation. Continuous monitoring and adaptive management enabled implementation bottlenecks to be identified and addressed during delivery, strengthening the practical relevance of the findings. Triangulation across quantitative, qualitative, adherence, market, supervision, and routine program data also enabled findings to be examined across multiple evidence streams.

The following limitations should be considered when interpreting the findings. First, implementation sites and qualitative informants were purposively selected, and sites had characteristics that supported implementation, including access to markets, vendors, connectivity, and financial services. Consequently, some findings may not be directly transferable to settings with weaker infrastructure, less-functional markets, or lower health-system capacity. This limitation was partly mitigated by examining operational challenges across implementation contexts and documenting adaptations made during delivery. Future implementation research should deliberately include more diverse sites to assess transferability under routine and lower-capacity conditions.

Second, the analysis relied substantially on program-generated monitoring records and operational documentation, which may have varied in completeness and may be subject to reporting or confirmation bias. Independent perspectives were also limited for some operational processes. We mitigated this limitation by triangulating program-generated records with qualitative assessments, adherence data, market assessments, supportive supervision, and program review records, and by retaining divergent findings rather than forcing a single interpretation. Future studies could strengthen independent assessment through external monitoring and additional independent qualitative inquiry.

Third, implementation procedures and delivery systems were adapted in response to emerging operational challenges. Although these adaptations were necessary to maintain service delivery, they introduced variation across districts and over time and may limit direct comparability between sites. Implementation conditions also varied according to season, market conditions, connectivity, staffing, and supervisory capacity. These factors were considered during interpretation by examining findings in relation to their timing and implementation context. Future studies should prospectively document adaptations, their rationale, timing, and consequences to enable more systematic assessment of how adaptation influences implementation fidelity and outcomes.

Fourth, this paper focuses on implementation experience and operational feasibility rather than clinical or economic effectiveness. Effectiveness and cost-effectiveness findings are reported separately and are therefore not presented here. Accordingly, the findings should not be interpreted as evidence of superiority, cost-effectiveness, or nutritional equivalence between unrestricted cash, locally sourced food vouchers, and SNF. Feasibility of delivery does not establish equivalent nutritional or clinical effects. Future analyses linking implementation characteristics with clinical, nutritional, and economic outcomes will be needed to address these questions.

Finally, several implementation inputs, including additional staffing, research and registration support, intensive supervision, technical assistance, and adaptive coordination, were available within the trial context and may not be routinely available during large-scale implementation. These supports may have contributed to the observed implementation performance and therefore limit direct extrapolation to routine programs. This limitation can be addressed through scale-up assessments that distinguish trial-specific resources from functions that can be integrated into existing systems. Programs considering scale-up should assess the workforce, market, digital, financial service, supervision, and coordination requirements of each modality and explicitly plan and budget for functions that cannot be absorbed within existing systems.

## 5. Conclusions

The implementation experience demonstrated that locally sourced food vouchers and unrestricted cash transfers can be operationally delivered within routine health system settings when supported by strong program preparation, market feasibility assessments, reliable digital and financial systems, continuous monitoring, and adaptive management. Modality selection should be based on local context, operational capacity, market functionality, and household conditions. Locally sourced food vouchers provided structured access to a predefined nutritious food basket while allowing adaptation to local food availability, but they required continuous market monitoring, retailer engagement, and quality assurance. Unrestricted cash provided greater household choice and flexibility but was more sensitive to payment systems, market conditions, price fluctuations, and household priorities and decision-making. Across both modalities, operational performance was shaped by workforce capacity, service accessibility, digital and financial system reliability, retailer compliance, accountability systems, and household-level constraints.

Overall, the trial generated practical implementation lessons on the conditions and systems required to deliver market-based nutrition interventions for MAM management. The findings highlight the importance of considering the interaction between modality design, health and market systems, and household realities when interpreting implementation experience and informing future program design and adaptation.

### 5.1. Recommendations for Replication in Other Contexts

Based on the implementation experience, the following considerations are particularly important when adapting market-based nutrition interventions to other similar settings:

#### 5.1.1. Government

• Develop minimum market-readiness criteria for introducing cash and voucher modalities, including food availability, retailer coverage, physical accessibility, price stability, and capacity to meet program demand.
• Integrate routine market monitoring into national MAM programming so that modality selection and program design can be adapted to differences across settings and over time.
• Establish standards for food quality, retailer compliance, beneficiary protection, and accountability within market-based nutrition programs.
• Incorporate the recurrent costs of digital systems, supervision, market monitoring, and workforce requirements into routine program planning and budgets when market-based approaches are taken to scale.

#### 5.1.2. Donors and Development Partners

• Invest not only in transfers, but also in the market infrastructure and systems required to deliver them, including retailer networks, digital systems, payment systems, and implementation capacity.
• Provide flexible resources that allow programs to respond to changes in food prices, availability, retailer capacity, and other market conditions.
• Support phased scale-up, with resources for readiness assessment, system strengthening, and adaptation before expansion.

#### 5.1.3. Implementing Agencies

• Conduct market assessments before implementation and establish routine mechanisms for monitoring food availability, prices, retailer performance, and beneficiary access.
• Strengthen retailer selection, training, supervision, and accountability to ensure that the intended food basket is consistently available and that beneficiaries can redeem their entitlements as intended.
• Integrate beneficiary support, SBCC, and feedback mechanisms into routine delivery, rather than treating them as additional activities.
• Establish practical contingency mechanisms for payment delays, digital system failures, retailer shortages, and physical access barriers.

#### 5.1.4. Financial and Digital Service Providers

• Ensure that payment and beneficiary management systems are reliable, timely, interoperable, and capable of functioning under low-connectivity conditions.
• Establish rapid mechanisms for resolving payment, registration, reconciliation, and synchronization problems that can interrupt access to assistance.

#### 5.1.5. Research and Technical Partners

• Generate continuous evidence on market readiness, food affordability and availability, dietary adequacy, adherence, implementation costs, and sustainability.

## Figures and Tables

**Figure 1:**
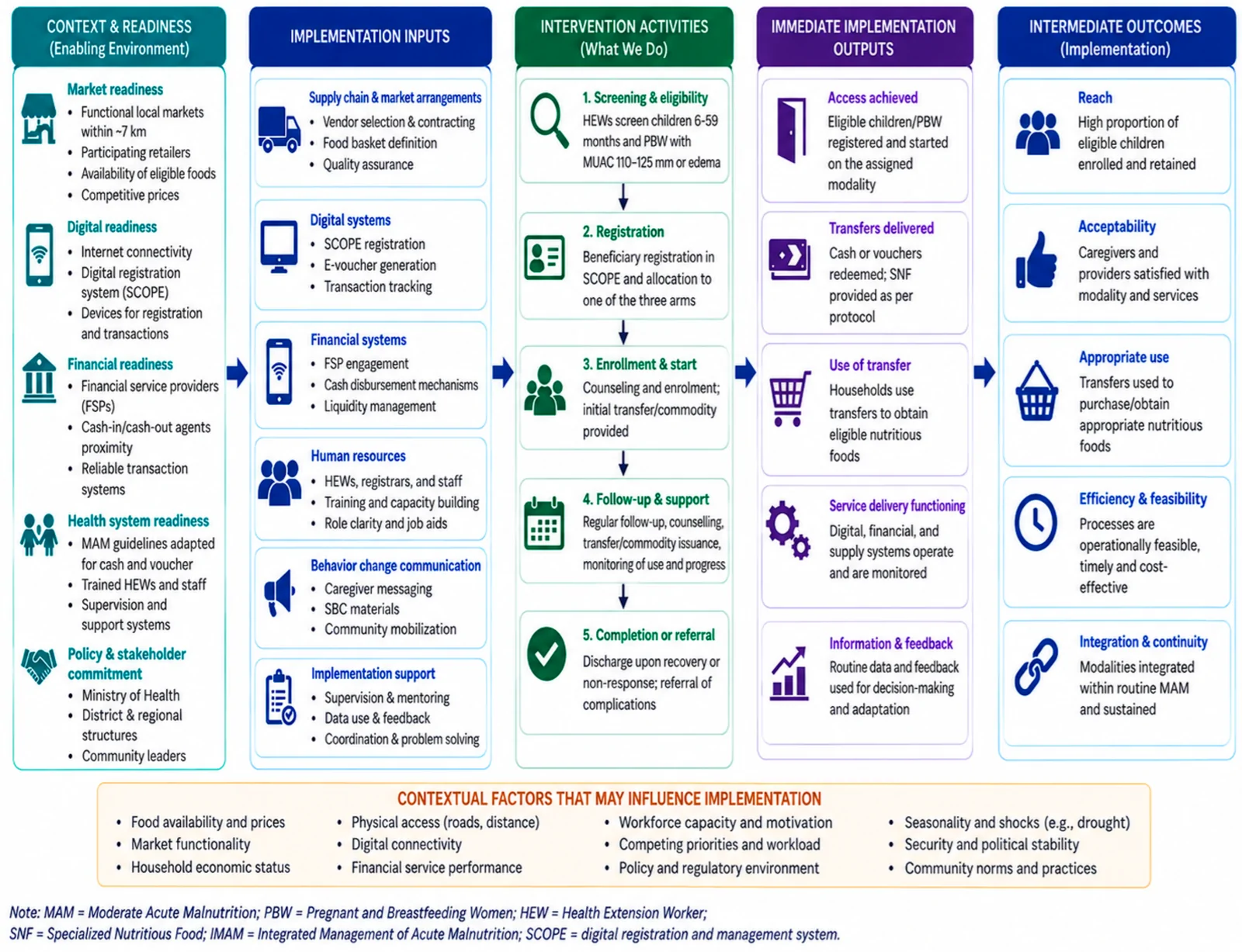
Implementation framework for food voucher and cash-based approaches for MAM management in Ethiopia.

**Figure 2:**
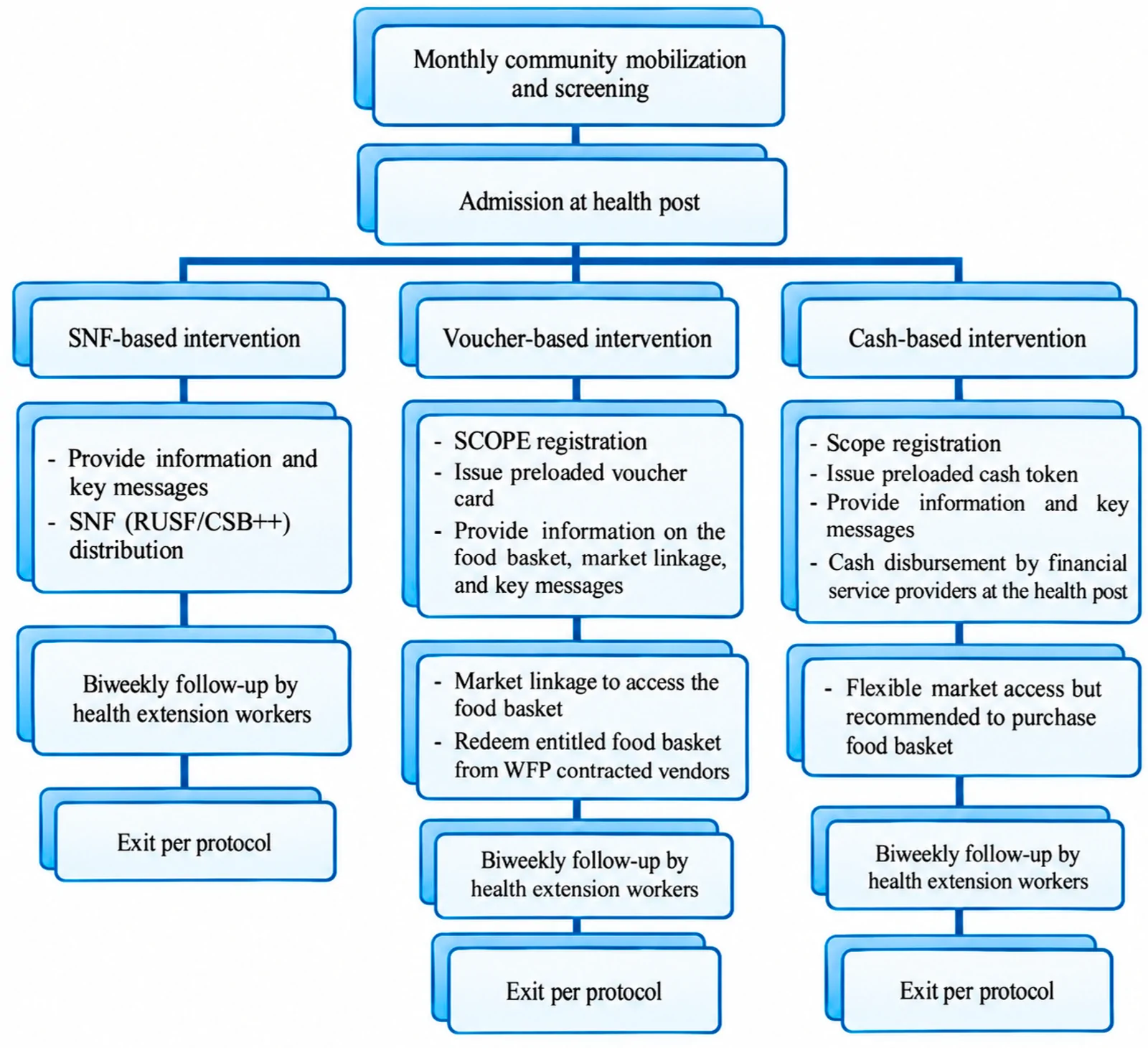
Process flow for specialized nutritious food (SNF), locally sourced food vouchers, and unrestricted cash transfers for the management of moderate acute malnutrition in Ethiopia.

**Table 1:** Key implementation actors and their roles in the VIMAM trial.

Level	Implementation Actor(s)	Primary Roles and Responsibilities
Community	Caregivers of children and Pregnant and breastfeeding women (PBW)	Primary participants and recipients of the intervention; attended nutrition screening, received cash/vouchers or SNF nutrition services, and participated in follow-up and SBCC activities.
	Kebele leaders, Religious leaders and community health development structures	Supported community mobilization, awareness creation and participant engagement
Health post	Health Extension Workers (HEWs)	Conducted nutrition screening and clinical assessments, enrollment, nutrition counseling, routine maternal and child health care services including treatment of identified medical conditions follow-up and referrals,
	Research assistants	Supported data collection, protocol implementation, verification of procedures, monitoring, and quality assurance.
	SCOPE registrars	Registered eligible participants in digital SCOPE platform, managed digital records, and tracked voucher/cash transactions.
	Financial Service Providers (FSPs) (both arms)	Facilitated on-site cash disbursement, transaction reconciliation, and secure, traceable financial flows
	WFP-contracted retailers (voucher arm)	Supplied recommended food baskets and redeemed vouchers while maintaining food availability and quality.
District	District nutrition focal persons, SCOPE coordinators, and district health offices	Coordinated implementation, provided supportive supervision and technical assistance, monitored program performance, and ensured adherence to national IMAM guidelines.
Regional	Regional Technical Advisory Groups	Provided strategic oversight, implementation support, technical guidance, and cross-site coordination.
National	Federal Ministry of Health (FMOH)	Provided policy leadership, regulatory oversight, and alignment with national nutrition policies and IMAM guidelines.
	World Food Programme (WFP) and implementing partners	Led operational coordination, supply chain management, SCOPE system support, market coordination, and overall program implementation.
	Addis Ababa University and Technical Advisory Groups	Provided scientific leadership, methodological guidance, implementation research oversight, and technical support.
	Data and Safety Monitoring Board (DSMB)	Independently monitored participant safety, data quality and integrity, trial conduct, and ethical compliance.

**Table 2:** Methodological characteristics and integration matrix of complementary data sources.

Evidence Source	Timing/Frequency	Data Source/Respondents	Sample	Purpose	Key Indicators	Evidence Type
Main effectiveness trial	During the trial intervention and follow-up period	Children aged 6–59 months and PBW enrolled in SNF, voucher, and cash arms	3296 children; 3340 PBW	Assess clinical and anthropometric outcomes and compare intervention arms	Clinical outcomes; anthropometric outcomes; treatment response; between-arm differences; non-inferiority outcomes	Quantitative
Adherence assessment	During the intervention follow-up period	Children/PBW and caregivers at household level	3044 children; 3071 PBW	Assess actual exposure, fidelity, utilization, and adherence to assigned interventions	Follow-up attendance; entitlement collection; voucher redemption; cash receipt; use and consumption; food sharing; SBCC exposure	Quantitative
Dietary assessment	During the defined dietary assessment period	Children and PBW	Approximately 290 children; 240 PBW	Assess dietary intake, dietary diversity, diet quality, and food sources across intervention modalities	Dietary diversity; nutrient intake; diet quality; food sources; consumption patterns	Quantitative
Qualitative implementation assessment	During the implementation period	Participants, caregivers, implementers, and key stakeholders	53 participants through FGDs, IDIs, and KIIs	Explore barriers, facilitators, experiences, and contextual factors affecting implementation	Operational barriers; facilitators; beneficiary and provider experiences; sociocultural factors; gender-related factors; system constraints; market barriers	Qualitative
Routine program monitoring and SCOPE records	Continuous throughout implementation	Beneficiaries, health posts, program records, and SCOPE transactions	Trial implementation sites (13 districts)	Monitor reach, participation, intervention delivery, transfer administration, and transactions	Registration; enrollment; follow-up; participation; entitlement delivery; voucher transactions; cash transfers; transaction completion and reconciliation	Quantitative/routine program data
Monthly supportive supervision	Monthly throughout implementation	Health posts, HEWs, research and implementation teams, WFP technical teams, district focal persons, and MOH representatives	All 13 districts	Assess implementation quality, fidelity, service delivery, and operational bottlenecks	Protocol adherence; intervention delivery; supply availability; documentation; beneficiary experience; service quality; operational challenges; corrective actions	Qualitative/structured monitoring
Market assessment	During implementation and through routine market monitoring	WFP-contracted vendors	245 of 668 contracted vendors	Assess market feasibility and factors affecting cash and voucher utilization	Food availability; prices and price trends; accessibility; vendor participation; compliance; preferences; market-support needs	Quantitative/market monitoring
Program review and coordination meetings	Throughout implementation	Program and implementation stakeholders	3 high-level coordination meetings; 15 meetings involving focal persons, MOH, and AAU	Identify recurring implementation issues, decisions, adaptations, and follow-up actions	Progress; implementation bottlenecks; action points; adaptations; coordination issues; unresolved challenges	Qualitative/documentary
Economic evaluation	During the economic evaluation period	Program and intervention cost records	Economic evaluation component	Estimate intervention costs, cost-effectiveness, and implications for scale-up	Intervention costs; resource requirements; cost-effectiveness; budget implications	Quantitative

**Table 3:** Triangulation framework for multi-source evidence integration.

Implementation Domain	Primary Evidence Sources	Complementary Sources	Triangulation and Synthesis Focus
Reach and participation	Routine monitoring; effectiveness trial	Adherence assessment; supervision	Participant enrollment, follow-up attendance, service continuity, and retention rates
Intervention fidelity and adherence	Adherence assessment; SCOPE records	Supervision; qualitative assessment	Adherence to protocol and whether interventions were received and utilized as intended
Voucher utilization and redemption	SCOPE records; adherence assessment	Adherence assessment; market assessment; supervision; qualitative assessment	Redemption dynamics, vendor accessibility, food availability, local pricing, and barriers to utilization
Cash transfer delivery and use	SCOPE records; adherence assessment	Supervision; qualitative assessment; meeting records	Receipt timeliness, cash access, liquidity, utilization patterns, and operational barriers
SBCC delivery	Routine monitoring; adherence assessment	Supervision; qualitative assessment	Caregiver exposure to group education, cooking demonstrations, and individual counseling
Market functionality	Market assessment	Voucher records; supervision; qualitative assessment	Local food availability, price trends, retailer compliance, hygiene, and accessibility
Operational systems	Supervision; routine monitoring; SCOPE records	Meeting minutes; qualitative assessment	Digital registration, payment processing, tablet synchronization, and staffing constraints
Adaptations and corrective actions	Supportive supervision	Meeting records; market monitoring; routine monitoring	Identification of system bottlenecks, corrective responses, and program adaptations
Beneficiary and provider experience	Qualitative assessment	Adherence assessment; supervision	Modality acceptability, perceived utility, household preferences, and systemic burdens

**Table 4:** Composition and estimated nutrient content of the 15-day voucher food basket for children aged 6–59 months and pregnant and breastfeeding women.

	Alternative	Food Items	Quantity (15 Days)	Energy (kcal)	Protein (g)	Fat (g)
Cereals	1	Barley	1.5 kg	5310	188	34.5
	1	Wheat	1.5 kg	5460	155	15
	2	Oats	1 kg	4040	147	91
	2	Maize	1 kg	5475	141	70.5
Pulses	1	Chickpea	1 kg	3640	193	6
	2	Peas	1 kg	3410	246	12
	3	Kidney bean	1 kg	3330	236	8
Oilseeds	1	Groundnut	0.5 kg	2835	129	246
	2	Flax seed	0.5 kg	2365	83.5	166
Vegetables	1	Carrot + kale	1 kg + 0.5 kg	525	24	4
	2	Carrot + spinach	1 kg + 0.5 kg	525	24	4
Fruit *	1	Avocado	2 kg	3200	40	294
	2	Mango	2 kg	1200	16	8
	2	Papaya	2 kg	860	10	6
Egg		Egg	15 (0.75 kg)	1073	94.5	71.2
Salt	NA	Iodised salt **	1 kg	0	0	0
Oil	NA	Cooking oil	0.25 kg	2210	0	250

* Fruit was an additional food group included only for pregnant and breastfeeding women to help meet their additional nutritional requirements. ** Iodized salt was provided once at admission for use throughout follow-up for both children and PBW. The estimated total 15-day ration provided 18,560 kcal, 681 g of protein, and 632 g of fat, corresponding to approximately 1237 kcal, 45 g of protein, and 42 g of fat per day. Nutrient values were estimated using standard food composition tables. NA not applicable. Note: alternative numbers indicate interchangeable options within each food group. One item from each alternative category was selected based on availability; alternatives were not intended to be provided cumulatively.

**Table 5:** Comparison of pre-load and post-registration voucher account management models.

Feature	Pre-Load Model	Post-Registration Model
Beneficiary identification	Caseload estimated by HEWs before beneficiary identification	Beneficiaries screened and eligibility confirmed before registration
SCOPE account creation	Temporary beneficiary (Dummy) profiles created in advance	Beneficiary accounts created after confirmed eligibility
Voucher entitlement/cash loading	Entitlements loaded before beneficiary registration	Entitlements loaded after registration
Linkage to beneficiary	Eligible beneficiaries linked to pre-loaded accounts	Beneficiaries directly registered and linked to their accounts
Access to first redemption	Immediate access after enrolment	Potential short delay depending on voucher or cash-cycle timing and system processing
Unused/pre-funded accounts	Risk of unused accounts when estimated caseload exceeded actual enrolment	Reduced risk because loading occurred after registration
Voucher/Cash numbering	Sequential numbering increased predictability	Non-sequential numbering used to strengthen security
Financial accountability	Weaker alignment between estimated caseload and actual enrolment	Better alignment between confirmed beneficiaries and voucher disbursement
Main operational advantage	Faster initial access to voucher/cash entitlements	Stronger registration and financial controls
Main limitation	Risk of idle accounts, unused balances, and potential misuse	Potential delay between eligibility confirmation and first redemption

**Table 6:** Summary of key implementation bottlenecks and lessons learned from implementation of the VIMAM trial in Ethiopia.

Implementation Domain	Key Bottleneck	Key Lesson Learned
Health workforce capacity	High workload, staff turnover, and competency gaps.	Adequate staffing, continuous capacity strengthening, and supportive supervision are essential.
Community engagement	Limited understanding of eligibility criteria and program processes.	Early and sustained community engagement supports program acceptance and targeting fidelity.
Facility and digital readiness	Connectivity, infrastructure, synchronization, and data recording challenges.	Digital delivery requires readiness assessment, reliable infrastructure, and contingency mechanisms.
Card and transfer supply chain	Voucher and cash card shortages	Supply planning should anticipate enrolment, treatment duration, and contingency needs.
Follow-up and data management	Incomplete follow-up, documentation gaps, and data inconsistencies.	Harmonized documentation, supervision, and routine data quality assurance are essential.
Retailer and market management	Price fluctuations, retailer non-compliance, and unauthorized substitutions.	Continuous retailer engagement, market monitoring, and accountability mechanisms are required.
Financial service delivery	Payment, payroll, reconciliation, and outreach challenges.	Financial service providers should be engaged as strategic implementation partners with clear roles and coordination mechanisms.
Caregiver and household factors	Distance, transport costs, storage limitations, perishability, and intra-household sharing.	Program design should account for practical access, storage, food utilization, and household constraints.
SBCC	Delays in materials, limited resources, and competing HEW responsibilities.	SBCC requires early planning, dedicated resources, standardized materials, and ongoing support.
Partner coordination	Inconsistent coordination and delayed resolution of operational issues.	Regular multi-stakeholder coordination strengthens ownership, accountability, and responsiveness.

## Data Availability

The qualitative data underlying the findings of this study are not publicly available because they contain potentially identifiable participant information. Relevant data may be available from the corresponding author on reasonable request, subject to ethical and confidentiality requirements.

## Supplementary Materials

The following supporting information can be downloaded at: https://www.mdpi.com/article/10.3390/nu18213633/s1. Table S1: Food Basket Price Values for Children Aged 6–59 Months and Pregnant and Breastfeeding Women by Region and Time Period in Ethiopia; Table S2: Cash Transfer Values for Children Aged 6–59 Months and Pregnant and Breastfeeding Women by Region and Time Period in Ethiopia; Table S3: Price Data for Alternative Food Baskets for Children Aged 6–59 Months Prior to Acceptability Testing; Table S4: Nutrient Profile for Alternative Food Baskets for Children Aged 6–59 Months Prior to Acceptability Testing; Table S5: Price Data for Alternative Food Baskets for Pregnant and Breastfeeding Women Prior to Acceptability Testing; Table S6: Nutrient Profile for Alternative Food Baskets for Pregnant and Breastfeeding Women Prior to Acceptability Testing; Table S7: Nutrient Profile For Final Food Baskets for Children Aged 6–59 Months after Acceptability Testing; Table S8: Nutrient Profile For Final Food Baskets for Pregnant and Breastfeeding Women after a Acceptability Testing; Table S9: Detailed Summary of Implementation Bottlenecks, Adaptive Management Actions, and Lessons Learned From Implementation of the VIMAM trial in Ethiopia.

## Abbreviations

AAU	Addis Ababa University.
CFM	Complaint and Feedback Mechanism
DSMB	Data Safety Monitoring Board
FMOH	Federal Ministry of Health
FSP	Financial Service Provider
HDA	Health Development Army
HEW	Health Extension Worker
IPC	Integrated Food Security Phase Classification
IYCF	Infant and Young Child Feeding
IMAM	Integrated Management of Acute Malnutrition
MAM	Moderate Acute Malnutrition
MSG	Market Support Group
MUAC	Mid-Upper Arm Circumference
PBW	Pregnant and Breastfeeding Women
PHCU	Primary Health Care Unit
SBCC	Social and Behavior Change Communication
SNF	Specialized Nutritious Food
TAC	Technical Advisory Committee
TSFP	Targeted Supplementary Feeding Program
VIMAM	Voucher and Cash Intervention for the Management of Moderate Acute Malnutrition
WFP	World Food Programme
WHO	World Health Organization

## References

[1] World Health Organization, United Nations Children’s Fund (UNICEF), World Bank Group (2025). Levels and Trends in Child Malnutrition: UNICEF/WHO/World Bank Group Joint Child Malnutrition Estimates: Key Findings of the 2025 Edition.

[2] World Health Organization (2023). WHO Guideline on the Prevention and Management of Wasting and Nutritional Oedema (Acute Malnutrition) in Infants and Children Under 5 Years.

[3] United Nations Children’s Fund (UNICEF) (2023). Undernourished and Overlooked: A Global Nutrition Crisis in Adolescent Girls and Women.

[4] Desyibelew H.D., Dadi A.F. (2019). Burden and Determinants of Malnutrition among Pregnant Women in Africa: A Systematic Review and Meta-Analysis. PLoS ONE.

[5] Black R.E., Victora C.G., Walker S.P., Bhutta Z.A., Christian P., de Onis M., Ezzati M., Grantham-McGregor S., Katz J., Martorell R. (2013). Maternal and Child Undernutrition and Overweight in Low-Income and Middle-Income Countries. Lancet.

[6] Ethiopian Statistical Service (ESS), ICF (2025). Ethiopia Demographic and Health Survey 2024–25: Key Indicators.

[7] OCHA (2024). Ethiopia—Situation Report. https://www.unocha.org/publications/report/ethiopia/ethiopia-situation-report-1-mar-2024.

[8] Grobler-Tanner C., Collins S. (2004). Community Therapeutic Care (CTC): A New Approach to Managing Acute Malnutrition in Emergencies and Beyond.

[9] Deconinck H., Swindale A., Grant F., Navarro-Colorado C. (2008). Review of Community-Based Management of Acute Malnutrition (CMAM) in the Post-Emergency Context: Synthesis of Lessons on Integration of CMAM into National Health Systems.

[10] Durao S., Visser M.E., Ramokolo V., Oliveira J.M., Schmidt B.-M., Balakrishna Y., Brand A., Kristjansson E., Schoonees A. (2020). Community-Level Interventions for Improving Access to Food in Low- and Middle-Income Countries. Cochrane Database Syst. Rev..

[11] Girard A.W., Olude O. (2012). Nutrition Education and Counselling Provided during Pregnancy: Effects on Maternal, Neonatal and Child Health Outcomes. Paediatr. Perinat. Epidemiol..

[12] Langlois B.K., Cliffer I.R., Nikiema L., Suri D.J., Garanet F., Shen Y., Zeba A.N., Walton S.M., Lanou H.B., Webb P. (2020). Factors That May Influence the Effectiveness of 4 Specialized Nutritious Foods in the Prevention of Stunting and Wasting in Children Aged 6–23 Months in Burkina Faso. Curr. Dev. Nutr..

[13] Bazzano A.N., Potts K.S., Bazzano L.A., Mason J.B. (2017). The Life Course Implications of Ready-to-Use Therapeutic Food for Children in Low-Income Countries. Int. J. Environ. Res. Public Health.

[14] WFP WFP Ethiopia Country Brief, 2025. https://www.wfp.org/countries/ethiopia.

[15] Collins S., Dent N., Binnns P., Bahwere P., Sadler K., Hallam A. (2006). Management of severe acute malnutrition in children. Lancet.

[16] FEWS NET (2025). Ethiopia Food Security Outlook Update, December 2025 to May 2026: Extended and Extreme Dry Conditions in the South Continue to Drive High Assistance Needs.

[17] A Synthesis of Lessons: Government Experiences of Scale-Up of Community-Based Management of Acute Malnutrition (CMAM)-Ethiopia|ReliefWeb. https://reliefweb.int/report/ethiopia/synthesis-lessons-government-experiences-scale-community-based-management-acute.

[18] Management of Severe Acute Malnutrition in Children-PubMed. https://pubmed.ncbi.nlm.nih.gov/17141707/.

[19] Kassahun E.A., Hassen H.Y., Farah A.M., Nigatu Y.D., Nane D., Keats E.C., Paulos G.K., Sissay T.A., Barsenga K., Kebede A. (2026). Local Food Interventions for the Management of Moderate Acute Malnutrition (MAM) among Children Aged 6 to 59 Months: A Systematic Review and Non-Inferiority Meta-Analysis. Adv. Nutr..

[20] WFP Evidence Summary: Cash-Based Transfers-Lessons from Evaluations-World|ReliefWeb. https://reliefweb.int/report/world/wfp-evidence-summary-cash-based-transfers-lessons-evaluations.

[21] Doocy S., Tappis H. (2017). Cash-Based Approaches in Humanitarian Emergencies: A Systematic Review. Campbell Syst. Rev. Better Evid. A Better World.

[22] López-Ejeda N., Charle Cuellar P., Vargas A., Guerrero S. (2018). Can Community Health Workers Manage Uncomplicated Severe Acute Malnutrition? A Review of Operational Experiences in Delivering Severe Acute Malnutrition Treatment through Community Health Platforms. Matern. Child Nutr..

[23] Taye T., Gebreyesus S.H., Farah A.M., Desalegn Y., Spigt M., Kebede A., Yilma B., Ackatia-Armah R., Barsenga K. (2026). How Well Is Ethiopia’s Moderate Acute Malnutrition Program Implemented?. Curr. Dev. Nutr..

[24] The Sphere Handbook: Humanitarian Charter and Minimum Standards in Humanitarian Response-2018 Edition-World|ReliefWeb. https://reliefweb.int/report/world/sphere-handbook-humanitarian-charter-and-minimum-standards-humanitarian-response-2018.

[25] Dad F., Dibari F., Kebede A., Lefu E., Ndumiyana T., Butaumocho B. (2023). Digitalisation in the WFP Fresh Food Voucher Programme: A Pilot Study from Rural Amhara Region, Ethiopia. Front. Nutr..

[26] Schuler F., Bitar J., Uekermann F., Taki M., Saidi M., Al Omran S., Choufari B., Meerkatt H. (2017). Food-Restricted Voucher or Unrestricted Cash? How to Best Support Syrian Refugees in Jordan and Lebanon.

[27] Teta I., Foudjo B.U.S., Nielsen J.N., Oben J., Nguefack-Tsague G., Ntentie F.R., Cakir V., Klemm R., Kang Y. (2023). Outcomes of a Food Voucher Program and Factors Associated with the Recovery Rate of Children with Moderate Acute Malnutrition in Far North Cameroon. J. Health Popul. Nutr..

[28] Hedlund K. (2012). Emerging Good Practice in the Use of Fresh Food Vouchers.

[29] Turk T., Paul R., Safdar N.F., Shah N., Alam S.M., Choudhury S.R., Shafique K., Ahmer Z., Wenndt A., Babar H.K. (2025). Integrating Co-Design into Formative Research for a SBCC Entry-Point Platform for Nutrition-Sensitive Social Protection Programs in Low -and Middle-Income Country Settings. PLoS ONE.

[30] Suchdev P.S., Jefferds M.E.D., Ota E., Da Silva Lopes K., De-Regil L.M. (2020). Home Fortification of Foods with Multiple Micronutrient Powders for Health and Nutrition in Children under Two Years of Age. Cochrane Database Syst. Rev..

