# Supplementary material for: Implementing Locally Sourced Food Vouchers and Unrestricted Cash Transfers for Moderate Acute Malnutrition Management in Ethiopia: Implementation Lessons from the VIMAM Trial
Source: Nutrients. 2026 Oct 31;18(21):3633. doi: 10.3390/nu18213633 (PMC13765230; doi:10.3390/nu18213633)
Supplement: Supplementary file 1 [file nutrients-18-03633-s001.zip › Table S1.pdf]

Table S1: Food Basket Price Values for Children Aged 6–59 Months and Pregnant and Breastfeeding Women by Region and Time Period in Ethiopia

**Population Group**	**Region**	**Food group**	**Approved quantity**	**Alternative 1**	**Alternative 2**	**Initial (ETB)**	**Revised (ETB)**
**Children (6–59 months)**	**South, Sidama & Central**	**Cereal**	2.5–3 kg	Barley (1.5 kg) + Oats (1 kg)	Wheat (1.5 kg) + Corn (1 kg)	380	500
		**Pulse**	1–1.5 kg	Pea (1 kg) / Chickpea (1 kg) / Kidney bean (1 kg)	—	202	300
		**Oilseed**	0.25–0.5 kg	Flaxseed (0.25 kg) / Groundnut (0.5 kg)	—	—	—
		**Vegetable**	1.5 kg	Carrot (1 kg) + Kale (0.5 kg)	—	60	100
		**Egg**	14–16	15 eggs	—	290	320
		**Cooking oil**	0.25 litre	Oil (0.25 litre)	—	80	120
		**Salt**	1 kg	Salt (1 kg)	—	—	—
		**Total basket value**				**1,012**	**1,340**
**Children (6–59 months)**	**Oromia**	**Cereal**	2.5–3 kg	Barley (1.5 kg) + Oats (1 kg)	Wheat (1.5 kg) + Corn (1 kg)	282.45	600
		**Pulse**	1–1.5 kg	Pea (1 kg) / Chickpea (1 kg) / Kidney bean (1 kg)	—	201.25	300
		**Oilseed**	0.25–0.5 kg	Flaxseed (0.25 kg) / Groundnut (0.5 kg)	—	—	—
		**Vegetable**	1.5 kg	Carrot (1 kg) + Kale (0.5 kg)	—	103.53	120
		**Egg**	14–16	15 eggs	—	267.75	275
		**Cooking oil**	0.25 litre	Oil (0.25 litre)	—	73.76	90
		**Salt**	1 kg	Salt (1 kg)	—	—	—
		**Total basket value**				**928.75**	**1,385**
**Pregnant & Breastfeeding Women**	**South, Sidama & Central**	**Cereal**	2.5–3 kg	Barley (1.5 kg) + Oats (1 kg)	Wheat (1.5 kg) + Corn (1 kg)	380	500
		**Pulse**	1–1.5 kg	Pea (1 kg) / Chickpea (1 kg) / Kidney bean (1 kg)	—	202	300
		**Oilseed**	0.25–0.5 kg	Flaxseed (0.25 kg) / Groundnut (0.5 kg)	—	—	—
		**Vegetable**	1.5 kg	Carrot (1 kg) + Kale (0.5 kg)	—	60	100

Population Group	Region	Food group	Approved quantity	Alternative 1	Alternative 2	Initial (ETB)	Revised (ETB)
		**Fruit**	2 kg	Avocado / Mango / Papaya (2 kg)	—	180	320
		**Egg**	14–16	15 eggs	—	290	250
		**Cooking oil**	0.25 litre	Oil (0.25 litre)	—	80	120
		**Salt**	1 kg	Salt (1 kg)	—	—	—
		**Total basket value**				**1,192**	**1,590**
**Pregnant & Breastfeeding Women**	**Oromia**	**Cereal**	2.5–3 kg	Barley (1.5 kg) + Oats (1 kg)	Wheat (1.5 kg) + Corn (1 kg)	282.45	600
		**Pulse**	1–1.5 kg	Pea (1 kg) / Chickpea (1 kg) / Kidney bean (1 kg)	—	201.25	300
		**Vegetable**	1.5 kg	Carrot (1 kg) + Kale (0.5 kg)	—	103.53	120
		**Fruit**	2 kg	Avocado / Mango / Papaya (2 kg)	—	252	300
		**Egg**	14–16	15 eggs	—	267.75	275
		**Cooking oil**	0.25 litre	Oil (0.25 litre)	—	73.76	90
		**Salt**	1 kg	Salt (1 kg)	—	—	—
		**Total basket value**				**1,180.75**	**1,685**

Note: PBW = Pregnant & Breastfeeding Women. Alternative 1 and Alternative 2 are interchangeable food combinations providing the approved quantity for that food group. "—" indicates that a price was not provided in the source data.
