# Supplementary material for: Implementing Locally Sourced Food Vouchers and Unrestricted Cash Transfers for Moderate Acute Malnutrition Management in Ethiopia: Implementation Lessons from the VIMAM Trial
Source: Nutrients. 2026 Oct 31;18(21):3633. doi: 10.3390/nu18213633 (PMC13765230; doi:10.3390/nu18213633)
Supplement: Supplementary file 1 [file nutrients-18-03633-s001.zip › Table S2.pdf]

Table S2: Cash Transfer Values for Children Aged 6–59 Months and Pregnant and Breastfeeding Women by Region and Time Period in Ethiopia

**Beneficiary group**	**Region**	**Basket value at program initiation, January 2025 (ETB)**	**Revised basket value, December 2025 (ETB)**
Children 6–59 months	South, Sidama & Central	1,012.00	1,340.00
Children 6–59 months	Oromia	928.75	1,385.00
PBW	South, Sidama & Central	1,192.00	1,590.00
PBW	Oromia	1,180.75	1,685.00

Note: PBW = pregnant and breastfeeding women. The initial basket value represents the estimated 15-day basket cost at program initiation in January 2025, while the revised basket value represents the estimated cost based on prices observed toward the end of program implementation in December 2025.
