# Supplementary material for: Implementing Locally Sourced Food Vouchers and Unrestricted Cash Transfers for Moderate Acute Malnutrition Management in Ethiopia: Implementation Lessons from the VIMAM Trial
Source: Nutrients. 2026 Oct 31;18(21):3633. doi: 10.3390/nu18213633 (PMC13765230; doi:10.3390/nu18213633)
Supplement: Supplementary file 1 [file nutrients-18-03633-s001.zip › Table S3.pdf]

Table S3: Price Data for Alternative Food Baskets for Children Aged 6–59 Months Prior to Acceptability Testing.

	Food Item	Price per 100g	Quantity in grams	Proportion of basket (in terms of grams)	total cost
Basket 1	Teff	7.66	125	29%	9.575
	Navy Bean	4.48	75	18%	3.36
	Egg	20.77	50	12%	10.385
	Leaf, collards, raw	2.83	100	24%	2.83
	Banana	6.83	50	12%	3.415
	Oil	14.82	25	6%	3.705
			425		**33.27**
Basket 2		**Price per 100g**	**Quantity to model**	**Proportion of basket (in terms of grams)**	**total cost**
	Teff	7.66	125	29%	9.575
	Bengal gram (chickpea)	5.02	75	18%	3.765
	Eggs	20.77	50	12%	10.385
	Carrot	3.95	100	24%	3.95
	Banana	6.83	50	12%	3.415
	Oil	14.82	25	6%	3.705
			425		**34.795**
Basket 3		**Price per 100g**	**Quantity to model**	**Proportion of basket (in terms of grams)**	**total cost**
	Whole grain wheat	4.81	125	29%	6.0125
	Navy bean	4.48	75	18%	3.36
	Egg	20.77	50	12%	10.385
	Carrot	3.95	100	24%	3.95
	Banana	6.83	50	12%	3.415
	Oil	14.82	25	6%	3.705
			425		**30.8275**
Basket 4		**Price per 100g**	**Quantity to model**	**Proportion of basket (in terms of grams)**	**total cost**
	Whole grain wheat	4.81	125	26%	6.0125
	Navy bean	4.48	75	16%	3.36
	Egg	20.77	50	11%	10.385
	Leaf, collards, raw	2.83	100	21%	2.83
	Carrot	3.95	100	21%	3.95
	Oil	14.82	25	5%	3.705
			475		**30.2425**
Basket 5		**Price per 100g**	**Quantity to model**	**Proportion of basket (in terms of grams)**	**total cost**
	Teff	7.66	125	26%	9.575
	Bengal gram (Chickpea)	5.02	75	16%	3.765
	Pumpkin	2.12	100	21%	2.12
	Leaf, collards	2.83	100	21%	2.83
	Egg	20.77	50	11%	10.385
	Oil	14.82	25	5%	3.705
			475		**32.38**
