# Supplementary material for: Implementing Locally Sourced Food Vouchers and Unrestricted Cash Transfers for Moderate Acute Malnutrition Management in Ethiopia: Implementation Lessons from the VIMAM Trial
Source: Nutrients. 2026 Oct 31;18(21):3633. doi: 10.3390/nu18213633 (PMC13765230; doi:10.3390/nu18213633)
Supplement: Supplementary file 1 [file nutrients-18-03633-s001.zip › Table S4.pdf]

Table S4: Nutrient Profile for Alternative Food Baskets for Children Aged 6–59 Months Prior to Acceptability Testing.

4 year Child	Energy	Protein	Fat	Vitamin A	Vitamin C	Vitamin B1	Vitamin B2	Niacin	Vitamin B6	Pantothenic Acid	Folic Acid	Vitamin B12	Calcium	Iron	Magnesium	Zinc	Average performance
**Basket 1 - 425g**	77%	127%	63%	31%	37%	142%	74%	60%	122%	74%	133%	29%	67%	148%	145%	45%	**86%**
**Basket 2 - 425g**	78%	117%	69%	53%	9%	96%	74%	75%	134%	68%	64%	29%	53%	168%	128%	41%	**78%**
**Basket 3 - 425g**	73%	117%	62%	53%	9%	151%	47%	81%	104%	79%	127%	29%	25%	118%	120%	32%	**77%**
**Basket 4 - 475g**	73%	123%	63%	68%	32%	153%	53%	83%	98%	82%	157%	29%	46%	120%	124%	32%	**83%**
**Basket 5 - 475g**	76%	123%	70%	42%	42%	100%	80%	78%	113%	70%	89%	29%	75%	169%	131%	41%	**83%**
**RUSF - 100g**	37%	35%	67%	64%	66%	124%	192%	114%	165%	110%	111%	196%	67%	83%	56%	62%	**97%**
