# Supplementary material for: Implementing Locally Sourced Food Vouchers and Unrestricted Cash Transfers for Moderate Acute Malnutrition Management in Ethiopia: Implementation Lessons from the VIMAM Trial
Source: Nutrients. 2026 Oct 31;18(21):3633. doi: 10.3390/nu18213633 (PMC13765230; doi:10.3390/nu18213633)
Supplement: Supplementary file 1 [file nutrients-18-03633-s001.zip › Table S5.pdf]

Table S5: Price Data for Alternative Food Baskets for Pregnant and Breastfeeding Women Prior to Acceptability Testing.

	Food Item	Price per 100g	Quantity in grams	Proportion of basket (in terms of grams)	total cost
Basket 1	Teff	7.66	200	0.32	15.32
	Navy Bean	4.48	100	0.16	4.48
	Egg	20.77	100	0.16	20.77
	Leaf, collards, raw	2.83	100	0.16	2.83
	Banana	6.83	100	0.16	6.83
	Oil	14.82	25	0.04	3.705
			625		**53.935**
Basket 2		Price per 100g	Quantity to model	Proportion of basket (in terms of grams)	total cost
	Teff	7.66	200	0.32	15.32
	Bengal gram (chickpea)	5.02	100	0.16	5.02
	Eggs	20.77	100	0.16	20.77
	Carrot	3.95	100	0.16	3.95
	Banana	6.83	100	0.16	6.83
	Oil	14.82	25	0.04	3.705
			625		**55.595**
Basket 3		Price per 100g	Quantity to model	Proportion of basket (in terms of grams)	total cost
	Whole grain wheat	4.81	200	0.32	9.62
	Navy bean	4.48	100	0.16	4.48
	Egg	20.77	100	0.16	20.77
	Carrot	3.95	100	0.16	3.95
	Banana	6.83	100	0.16	6.83
	Oil	14.82	25	0.04	3.705
			625		**49.355**
Basket 4		Price per 100g	Quantity to model	Proportion of basket (in terms of grams)	total cost
	Whole grain wheat	4.81	200	0.32	9.62
	Navy bean	4.48	100	0.16	4.48
	Egg	20.77	100	0.16	20.77
	Leaf, collards, raw	2.83	100	0.16	2.83
	Carrot	3.95	100	0.16	3.95
	Oil	14.82	25	0.04	3.705
			625		**45.355**
Basket 5		Price per 100g	Quantity to model	Proportion of basket (in terms of grams)	total cost
	Teff	7.66	200	0.32	15.32
	Bengal gram (Chickpea)	5.02	100	0.16	5.02
	Pumpkin	2.12	100	0.16	2.12
	Leaf, collards	2.83	100	0.16	2.83
	Egg	20.77	100	0.16	20.77
	Oil	14.82	25	0.04	3.705
			625		**49.765**
