# Supplementary material for: Implementing Locally Sourced Food Vouchers and Unrestricted Cash Transfers for Moderate Acute Malnutrition Management in Ethiopia: Implementation Lessons from the VIMAM Trial
Source: Nutrients. 2026 Oct 31;18(21):3633. doi: 10.3390/nu18213633 (PMC13765230; doi:10.3390/nu18213633)
Supplement: Supplementary file 1 [file nutrients-18-03633-s001.zip › Table S6.pdf]

Table S6: Nutrient Profile for Alternative Food Baskets for Pregnant and Breastfeeding Women Prior to Acceptability Testing.

Breastfeeding woman	Energy	Protein	Fat	Vitamin A	Vitamin C	Vitamin B1	Vitamin B2	Niacin	Vitamin B6	Pantothenic Acid	Folic Acid	Vitamin B12	Calcium	Iron	Magnesium	Zinc	Average performance
**Basket 1 - 465g**	51%	106%	35%	67%	73%	151%	116%	72%	159%	61%	129%	33%	74%	50%	220%	118%	**95%**
**Basket 2 - 465g**	51%	100%	39%	156%	28%	109%	120%	91%	173%	58%	67%	33%	65%	56%	200%	109%	**91%**
**Basket 3 - 465g**	48%	100%	34%	156%	28%	166%	79%	103%	135%	67%	134%	33%	29%	40%	184%	82%	**89%**
**Basket 4 - 485g**	47%	103%	35%	193%	73%	166%	82%	104%	113%	67%	155%	33%	46%	40%	184%	82%	**95%**
**Basket 5 - 485g**	50%	103%	39%	125%	97%	110%	123%	92%	140%	58%	84%	33%	83%	56%	199%	108%	**94%**
**Super Cereal Plus (CSB++) 250g**	35%	68%	20%	549%	518%	48%	320%	145%	194%	59%	70%	208%	105%	28%	35%	120%	**158%**
