# Supplementary material for: Implementing Locally Sourced Food Vouchers and Unrestricted Cash Transfers for Moderate Acute Malnutrition Management in Ethiopia: Implementation Lessons from the VIMAM Trial
Source: Nutrients. 2026 Oct 31;18(21):3633. doi: 10.3390/nu18213633 (PMC13765230; doi:10.3390/nu18213633)
Supplement: Supplementary file 1 [file nutrients-18-03633-s001.zip › Table S9.pdf]

Table S9: Detailed Summary of Implementation Bottlenecks, Adaptive Management Actions, and Lessons Learned From Implementation of the VIMAM trial in Ethiopia

Implementation domain	Phase	Key bottleneck	Adaptive management action	Key lesson learned
**Health workforce capacity and competency**	Early	High HEW workload, staff turnover, inadequate refresher training, and gaps in knowledge of voucher procedures, food basket contents, counselling, and documentation affected implementation consistency.	Refresher training, mentoring, supportive supervision, deployment of additional personnel, and closer coordination with facility teams were implemented.	New nutrition modalities increase frontline workload and require adequate staffing, continuous capacity strengthening, and supportive supervision.
**Community engagement and program acceptance**	Early	Community pressure for broader enrolment and limited understanding of eligibility criteria challenged adherence to targeting procedures.	Targeted community sensitization, coffee conversations, and engagement of HEWs, religious leaders, and other community stakeholders were strengthened.	Meaningful community engagement and dialogue are essential for acceptance of eligibility criteria and program processes.
**Facility and digital readiness**	Early	Variation in facility readiness, connectivity constraints, limited technical infrastructure, delayed synchronization, and inconsistencies in digital records affected registration and transfer processing.	Targeted technical support, decentralized troubleshooting, improved data reconciliation, and practical onsite solutions were introduced.	Digital delivery requires early readiness assessment, reliable infrastructure, contingency mechanisms, and responsive technical support.

**Card and transfer supply chain**	Early	Shortages of cash and voucher cards during high enrolment periods created participant waiting times and risked interruptions in service delivery.	Additional cards were procured and distributed based on updated enrolment projections and caseloads.	Card requirements should be based on projected enrolment, expected treatment duration, and contingency needs.
**Follow-up and data management**	Throughout implementation	Poor adherence to follow-up schedules, incomplete documentation, weak defaulter tracking, and discrepancies between SCOPE and HEW records affected continuity and data quality.	Supportive supervision, routine data quality assessments, strengthened coordination, and improved defaulter tracing were implemented.	Harmonized documentation, continuous supervision, and routine data quality assurance are essential for implementation fidelity and retention.
**Retailer and market management**	Throughout implementation	Retailer knowledge gaps, market fluctuations, non-compliance, incomplete baskets, unauthorized substitutions, and weak market oversight affected voucher implementation.	Refresher training, retailer supervision, strengthened Market Support Groups, joint market monitoring, and corrective actions were implemented.	Voucher programs require continuous retailer engagement, active market oversight, and clear accountability mechanisms.
**Financial service delivery**	Throughout implementation	Payment delays, token mismatches, payroll and reconciliation errors, limited FSP familiarity with program procedures, and challenges reaching remote health posts affected payment continuity.	Regular coordination, weekly reconciliation, technical support, clearer roles, improved outreach arrangements, and strengthened FSP staffing were implemented.	FSPs should be engaged as strategic implementation partners, with early planning, adequate staffing, clear logistics, and routine coordination.

**Caregiver access and household constraints**	Throughout implementation	Long distances to retailers, transport costs, limited household storage, perishability of some foods, and intra-household sharing affected timely and appropriate use of the food basket.	Retailer coverage was expanded, caregiver counselling and HEW home visits were strengthened, and guidance on food handling and utilization was provided.	Food basket design and service arrangements should consider household storage, perishability, accessibility, transport costs, and household dynamics.
**SBCC**	Throughout implementation	Delays in SBCC materials, limited resources for cooking demonstrations, and competing HEW responsibilities reduced the consistency of some activities.	Flexible scheduling, use of locally available ingredients, provision of SBCC materials, mentoring, and supportive supervision were used.	SBCC requires early planning, dedicated resources, standardized materials, and integration into routine implementation.
**Partner coordination**	Throughout implementation	Limited coordination and variation in engagement among implementing partners affected timely identification and resolution of operational bottlenecks.	Regular coordination meetings, defined responsibilities, routine review of implementation progress, and action tracking were established.	Regular multi-stakeholder coordination strengthens ownership, accountability, and timely problem solving.
